# Superactivation of AMPA receptors by auxiliary proteins

**DOI:** 10.1038/ncomms10178

**Published:** 2016-01-08

**Authors:** Anna L. Carbone, Andrew J. R. Plested

**Affiliations:** 1 Leibniz-Institut für Molekulare Pharmakologie (FMP), Robert-Rössle-Straβe 10, 13125 Berlin, Germany; 2 NeuroCure, Charité-Universitätsmedizin Berlin, Charitéplatz 1, 10117 Berlin, Germany

## Abstract

Glutamate receptors form complexes in the brain with auxiliary proteins, which control their activity during fast synaptic transmission through a seemingly bewildering array of effects. Here we devise a way to isolate the activation of complexes using polyamines, which enables us to show that transmembrane AMPA receptor regulatory proteins (TARPs) exert their effects principally on the channel opening reaction. A thermodynamic argument suggests that because TARPs promote channel opening, receptor activation promotes AMPAR-TARP complexes into a superactive state with high open probability. A simple model based on this idea predicts all known effects of TARPs on AMPA receptor function. This model also predicts unexpected phenomena including massive potentiation in the absence of desensitization and supramaximal recovery that we subsequently detected in electrophysiological recordings. This transient positive feedback mechanism has implications for information processing in the brain, because it should allow activity-dependent facilitation of excitatory synaptic transmission through a postsynaptic mechanism.

## Abstract (web summary)

Transmembrane AMPA receptor regulatory proteins (TARPs) exert a number of effects on fast glutamatergic synaptic transmission. Here, the authors model the interactions of two such proteins, Stargazin and γ-8, and propose a simple kinetic mechanism by which TARPs modulate channel opening reaction.

## Graphical abstract

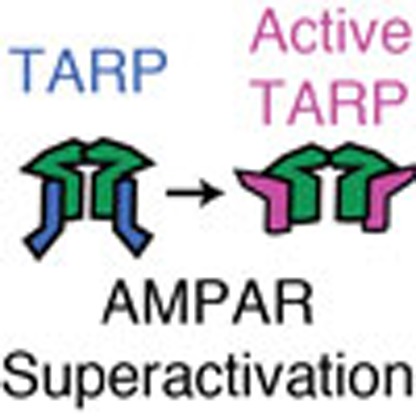

AMPA-type glutamate receptors (AMPARs) mediate the vast majority of the fast excitatory transmission in the brain. Like most ion channels, native AMPARs are macromolecular complexes of GluA subunits and accessory proteins. Stargazin, also known as γ-2, is the prototype auxiliary subunit of AMPARs^1^,^2^. Its absence in the Stargazer mouse provokes loss of functional synaptic receptors in cerebellar granule cells^2^,^3^ due to interplay between several transmembrane AMPA receptor regulatory proteins (TARPs)^4^. In addition to controlling the expression and the abundance of receptors at the postsynaptic membrane^2^,^5^,^6^, TARPs modulate the gating and pharmacology of AMPARs^7^,^8^. The kinetics of AMPARs expressed at synapses shape synaptic currents, and can determine short-term plasticity, over timescales far exceeding the glutamate transient from a single release event^9^. In principle, any kinetic transitions that are slow enough to allow AMPAR complexes to accumulate in different states during repetitive activity have implications for neuronal computation and sensory integration^10^.

To date, the models proposed to explain the apparently complex effects of Stargazin on AMPARs are inconsistent^8^,^11^,^12^,^13^. We devised a new method to isolate the activation of complexes, enabling us to detect the effects of Stargazin and another type-1 TARP (γ-8) that were previously missed. Using a series of AMPAR mutants, we developed a simple kinetic mechanism for AMPARs in complex with Stargazin. This mechanism predicts all reported behaviour of such complexes, and some previously unsuspected gating phenomena, establishing a general principle for TARP modulation of AMPAR function. We propose that TARPs exist in at least two different conformational states, and can themselves be activated by the receptor, with implications for synaptic transmission.

## Results

### Isolating complexes of AMPARs with Stargazin

**Figure 1:**
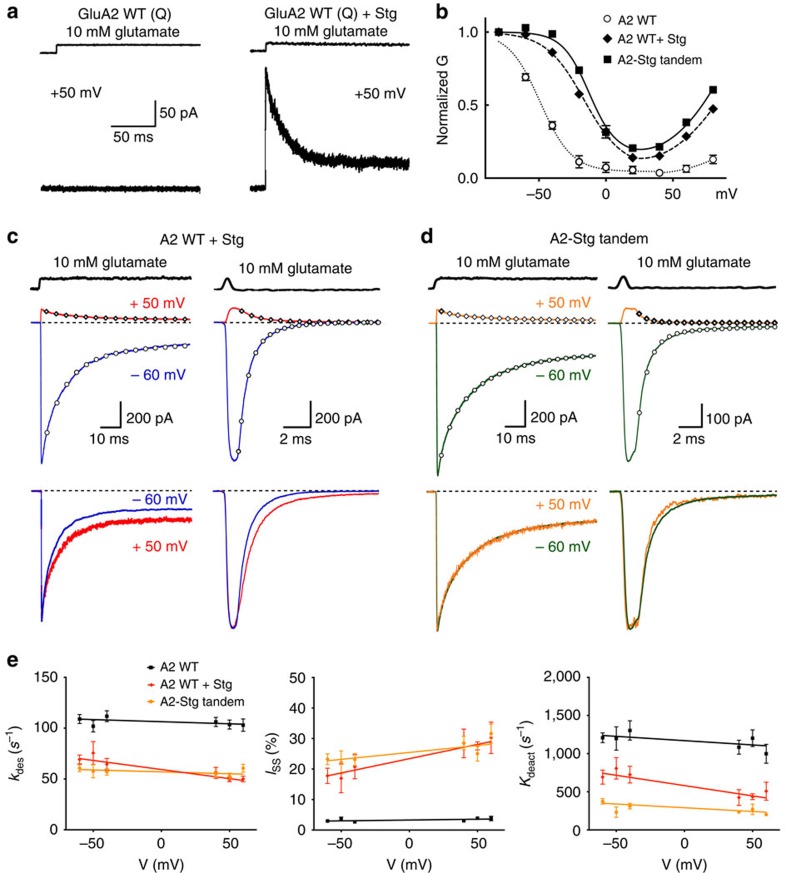
Isolating AMPAR-Stargazin complexes using intracellular polyamines. (**a**) Currents from GluA2 receptors alone were blocked by polyamines and responses were indistinguishable from the noise at +50 mV (left). Only cells coexpressing GluA2 and Stargazin showed a current at +50 mV in the presence of 50 μM intracellular spermine (right). (**b**) *G*–*V* relations in the presence of intracellular spermine for peak currents evoked by 10 mM glutamate for GluA2 alone (*n*=15), GluA2 cotransfected with Stargazin (*n*=9) and GluA2-Stargazin tandem (*n*=7). (**c**) Representative current responses from cells coexpressing GluA2 and Stargazin in response to 500 ms (left) and 1 ms (right) applications of glutamate recorded at +50 or −60 mV (with 50 μM intracellular spermine). Normalizing to the peak current revealed a greater apparent effect of Stargazin at +50 mV (bottom panels*; I*_SS_=20 and 12%; *k*_deact_=700 and 1300, s^−1^ at +50 mV and −60 mV, respectively). (**d**) Currents from cells expressing the GluA2-Stargazin tandem showed no voltage dependence (*I*_SS_=18%; *k*_deact_=1,100 and 1,000 s^−1^ at +50 mV and −60 mV, respectively). Representative solution exchange profiles are shown above the traces. (**e**) Voltage dependence of the rate of desensitization, the steady-state current and the deactivation rate of GluA2 cotransfected with Stargazin (red diamonds, *n*=10–44), GluA2-Stargazin tandem (orange circles, *n*=8–25) and GluA2 alone (black squares, *n*=10–30). Error bars represent s.e.m.

We coexpressed GluA2 homomeric receptors unedited at the Q/R site and Stargazin in HEK cells and recorded outside out patches including 50 μM spermine in the pipette. As previously shown^14^, in the presence of intracellular polyamines, only cells expressing both GluA2 and Stargazin showed current at +50 mV in response to 10 mM glutamate (150±15 pA, *n*=45). The response of cells transfected with GluA2 only was typically indistinguishable from the noise, because receptors that are not in complex with TARPs are blocked by intracellular polyamines (5±0.7 pA, *n*=15; Fig. 1a). However, *G*–*V* relations showed that an engineered GluA2-Stargazin tandem construct^15^,^16^ had more profound relief of polyamine block (Fig. 1b). This difference led us to hypothesize that in a simple cotransfection, corresponding to almost all previously published work, the expressed receptors were a mosaic mixture. In this mixture, some of the receptors were associated with fewer Stargazin molecules than others, and it is conceivable that some lacked any TARPs whatsoever. Consistent with this idea, Stargazin showed a larger apparent effect on the kinetics of GluA2 receptors at +50 mV, compared with –60 mV, presumably because of more profound voltage-dependent polyamine block in receptors with lesser TARP content (*k*_des_=50±2 and 70±4 s^−1^, *P*=0.00004, *n*=42 and 17; *k*_deact_=450±40 and 690±90 s^−1^, *P*=0.0044, *n*=44 and 13; *I*_ss_=30±2% and 15±2%, *P*=0.00001, *n*=39 and 16 for +50 and –60 mV, respectively; Fig. 1c). A full analysis over voltages from −60 to +60 mV is shown in Fig. 1e, Supplementary Fig. 1A,B and Supplementary Table 1. This voltage dependence was not observed in the absence of polyamine (Supplementary Fig. 1A,B and Supplementary Table 1). More profound differences with recording potential were observed for some of the mutants used in this study (Supplementary Fig. 1C). Moreover, some patches at –60 mV showed time-dependent increase in the desensitization rate and concurrent reduction in the steady-state current, possibly due to dynamic dissociation of Stargazin from complexes during the recording^16^. These time-dependent effects on kinetic properties were not observed at +50 mV (Supplementary Fig. 1D), presumably because only receptors with a heavy TARP content pass appreciable ionic current at this potential (Fig. 1b). In contrast, the kinetics of the GluA2-Stargazin tandem, and wild-type GluA2 in the absence of polyamine, were indistinguishable between positive and negative potentials (Fig. 1d,e; Supplementary Fig. 1A,B and Supplementary Table 1). These results exclude spurious voltage-dependent effects of Stargazin or voltage-dependent gating of AMPARs within complexes.

Although these data suggested that the currents we isolated at positive potential were comparatively rich in TARPs, we measured the efficacy of kainate relative to that of glutamate, to estimate the average TARP content of complexes^15^. At +50 mV, kainate efficacy from cells coexpressing GluA2 and Stargazin was indistinguishable from that of cells expressing the GluA2-Stargazin tandem (0.38±0.05, *n*=20 and 0.43±0.08, *n*=4 respectively, *P*=0.147, Supplementary Fig. 2). However, at –60 mV, KA/Glu ratio was much more variable, and on average less than in the tandem (0.28±0.14 and 0.35±0.03, *n*=12 and 6, respectively, *P*=0.0069, Supplementary Fig. 2). By this measure, in cells cotransfected with GluA2 and Stargazin, the complexes that we isolated at +50 mV were saturated with TARPs. These results further support our hypothesis that coexpression produces a variable, mixed population of receptors with different levels of TARP content, and possibly some receptors that lack TARPs altogether. Distinct from previously published work, in subsequent experiments we enriched for complexes with a higher TARP content, by working at +50 mV in the presence of intracellular polyamines. This measure mimics more closely the synaptic receptor population, where receptors are predominantly TARPed^15^. Even more importantly, by isolating complexes with a heavy TARP content in all the subsequent experiments we report here, we eliminated cell-to-cell variability from unequal expression levels, enabling us to monitor effects of TARPs that, in almost all previously published work, were diluted or essentially absent because of a variable, unquantified background of receptors either with few or no TARPs.

### State dependence of modulation by stargazin

**Figure 2:**
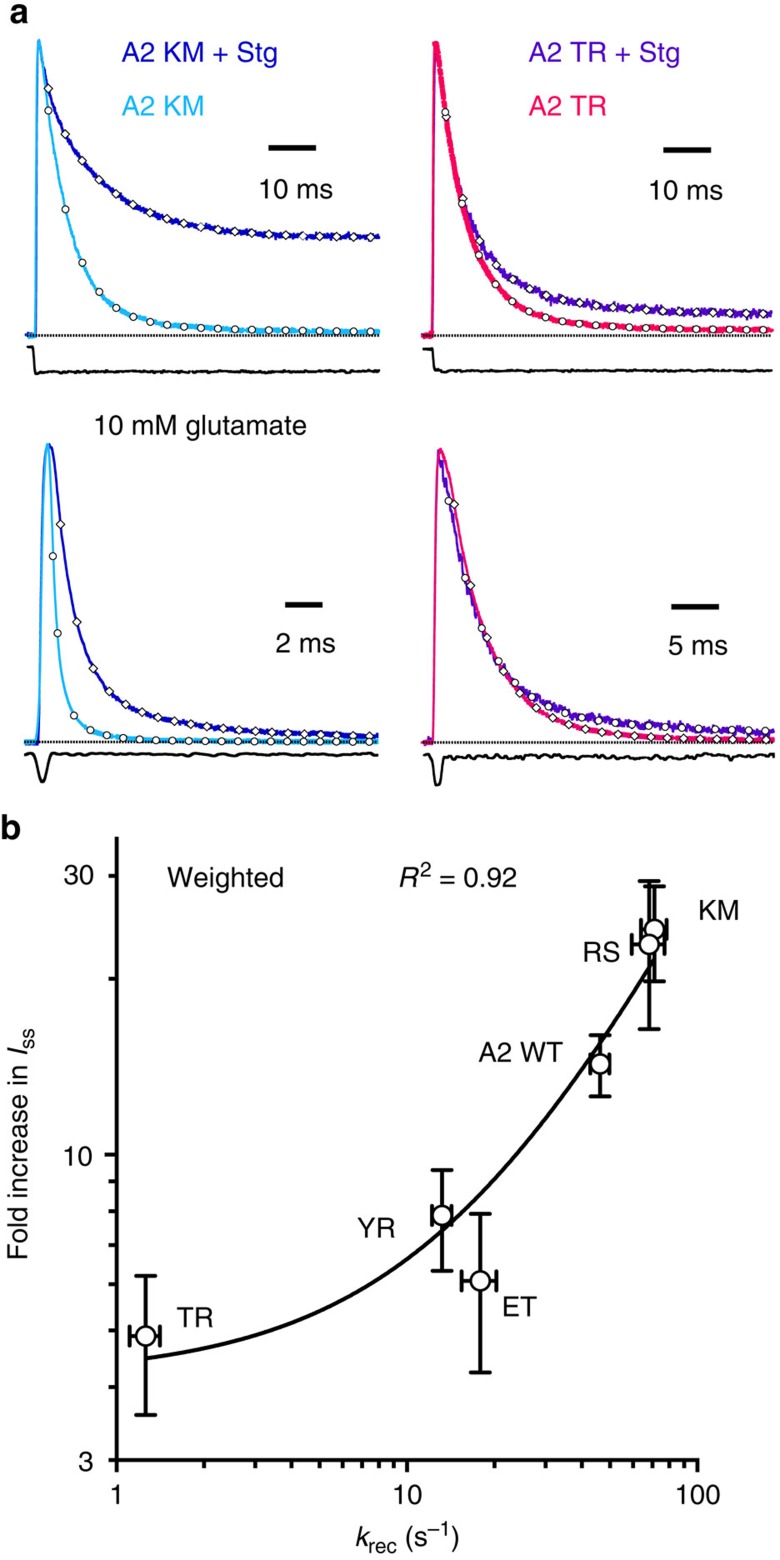
Kinetic effects of Stargazin are correlated to open state availability. (**a**) Representative traces from GluA2 K761M (left) and GluA2 E713T/Y768R (right) with and without Stargazin evoked by long (top) and short (bottom) applications of 10 mM glutamate. Stargazin significantly affected channel kinetics for the fast recovering mutant A2 K761M (*k*_des_=150 and 50 s^−1^; *I*_ss_=2 and 30%; *k*_deact_=3,100 and 860 s^−1^, in the example traces of receptors without and with Stargazin, respectively) but had less effect on the slow recovering mutant A2 E713T/Y768R (*k*_des_=170 and 160 s^−1^; *I*_ss_=1 and 7%; *k*_deact_=270 and 260 s^−1^, in these example traces without and with Stargazin, respectively). (**b**) A strong correlation was observed between the increase in the steady-state current induced by Stargazin and the recovery rate of the receptors (weighted *R*^2^=0.96, *n*=6–34). Fast recovering mutants showed at least 20-fold increase in the level of steady-state current when coexpressed with Stargazin; in contrast, mutants with slower recovery displayed only a modest increase in the steady-state current. Error bars represent s.e.m.

To investigate the mechanism of modulation of AMPAR gating by Stargazin, we used a panel of ligand-binding domain mutants, which spend very different fractions of time in the active versus desensitized states in saturating glutamate^17^ (Supplementary Fig. 3). All mutants tested showed similar relief of polyamine block, measured as an increase in the rectification index (*RI*=*I*_+40_/*I*_−60_, Supplementary Fig. 3C), suggesting that they were all able to form complexes with Stargazin. In any case, working at +50 mV removed any confounding effect of differential association of the mutants with Stargazin. Kinetic analysis of the mutants showed that the effect of Stargazin was correlated to the fraction of time that receptors spend in the desensitized state. Stargazin had limited effects on the kinetics of mutants with long-lived desensitized states, such as GluA2 E713T, GluA2 Y768R and GluA2 E713T/Y768R. However, coexpression with Stargazin massively increased the steady-state current for receptors with fast recovery (and thus more available open state), such as GluA2 R675S and GluA2 K761M (see Supplementary Table 2 and Fig. 2a). This increase in the steady-state current was strongly correlated to the rate of recovery from the desensitized state (*R*^*2*^=0.92; Fig. 2b, Supplementary Table 2). This correlation suggested that the potentiation of receptor gating by Stargazin is a positive feedback mechanism that depends on availability of the receptor in the open state.

### A mechanism for AMPAR-Stargazin complexes

**Figure 3:**
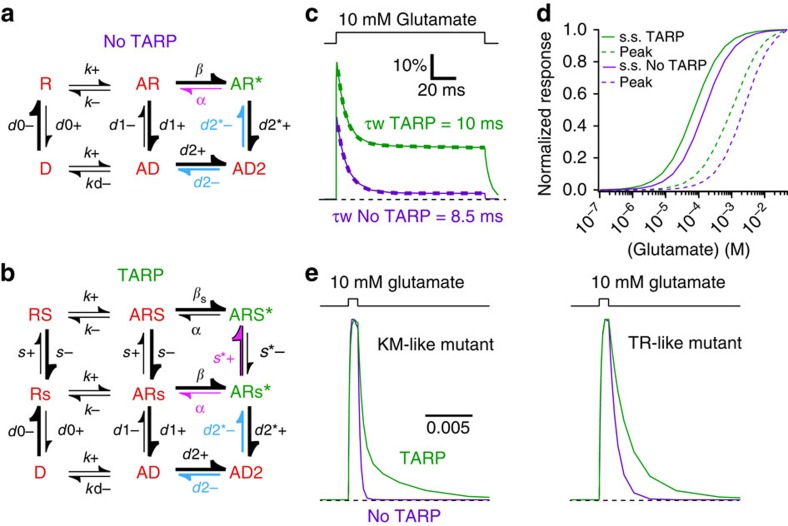
A model for Stargazin modulation. (**a**) Simplified single binding site model of AMPARs without TARPs. Open state is green, shut states are red. The kinetic behaviour of the mutant series was reproduced by altering the lifetime of the desensitized state AD2 (blue-arrowed rates), which was compensated by the channel shutting rate (*α*, pink) to maintain microscopic reversibility. (**b**) Model including TARPs, with boosted channel opening rate (*β*_s_) in the TARP-active open state only. Other rate constants were as in **a**. The rate of TARP activation (s*+, pink) compensated alterations to the channel shutting rate (*α*) over the mutant series, to maintain microscopic reversibility. (**c**) The TARP model predicted larger peak open probability and steady-state currents, and slower desensitization (Trial #7, see Methods). (**d**) Concentration response relations were left-shifted for the TARP model, despite equivalent binding rate constants. (**e**) The TARP model predicted the more profound slow component in the decay following a 1 ms pulse of glutamate for fast recovering mutants (compare with Fig. 2a).

We previously proposed a kinetic mechanism that recapitulates the broad spectrum of desensitization and gating behaviour of the series of ligand binding domain (LBD) mutants^17^ (Fig. 3a and Methods). We reasoned that this mechanism should also be able to describe the effects of Stargazin on these mutants, given appropriate modifications. We included extra states to represent the assumption that Stargazin itself can exist in a basal state that has no kinetic influence on the complex, and an active state in which the receptor has an increased channel opening rate and mean conductance (Fig. 3b). In resting and bound states, Stargazin was biased towards its basal state, but enforcing microscopic reversibility required that Stargazin be promoted towards its active state by channel opening (thus s*+ >> *s*^+^, see Methods). The open state with Stargazin active (ARS*) had 2.5-fold higher mean conductance than the basal open state (ARs*), reflecting greater average occupancy of high conductance states^8^,^18^,^19^. We presumed that glutamate binding rates and relief of polyamine block were identical, whether Stargazin was active or not. This simplified model predicted all known biophysical properties of Stargazin-containing complexes, including larger peak open probability and steady-state current, slower desensitization (Fig. 3c) and higher glutamate apparent affinity (Fig. 3d). Because about 5% of the population are already superactive in basal conditions (state RS), and are primed for opening to a high conductance, the mechanism also predicted the pronounced slow component in the deactivation decays for fast recovering mutants (Fig. 3e), and thus the lengthening of excitatory postsynaptic currents by TARPs. Finally, a modified version of the model predicted very well the effect of Stargazin on currents induced by kainate, and the increase in the KA/Glu current ratio, with only minimal modifications to allow for the weak partial agonism of kainate (Supplementary Fig. 4).

### Recovery overshoot

**Figure 4:**
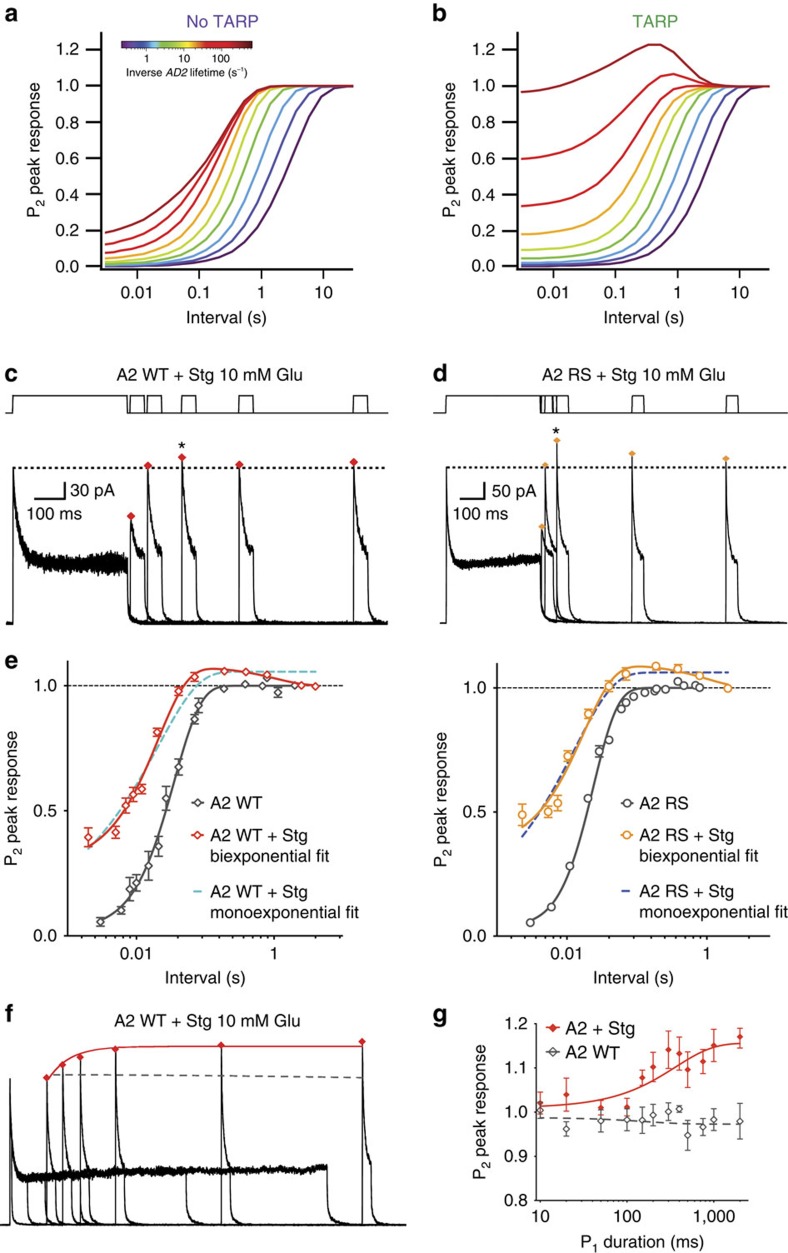
Stargazin induces suprarecovery without changing the rate of recovery from desensitization. (**a**) Simulated relaxations following equilibrium desensitization in 10 mM glutamate for the model in Fig. 3a. (**b**) The model from Fig. 3b predicted an overshoot in the recovery for fast recovering mutants. (**c**) During recovery, GluA2 WT+Stargazin currents recovered to a higher level than the initial pulse (red diamonds; for the patch shown, 107% of the initial value; *k*_rec_=40 s^−1^). (**d**) The overshoot was more profound for the GluA2 R675S mutant (in this example, 120% of the initial peak current; *k*_rec_=84 s^−1^. (**e**) Summary of recovery data for GluA2 WT and R675S. A monoexponential fit gave a faster rate of recovery in the presence of Stargazin, for both GluA2 WT (from 46±4 s^−1^, *n*=14 without Stargazin to 90±6 s^−1^ with Stargazin, *n*=25) and GluA2 R675S receptors (from 70±8 s^−1^, *n*=15–130±20 s^−1^, *n*=15). For biexponential fits, recovery rates with Stargazin were indistinguishable from those of receptors in the absence of Stargazin (53±3 and 70±8 s^−1^ for GluA2 WT and GluA2 R675S, respectively). The rates of dissipation of suprarecovery were: 0.95±0.2 and 2.1±0.7 s^−1^ for GluA2 WT (*n*=18) and R675S (*n*=15), respectively. (**f**) Increasing the length of the conditioning pulse (from 10 to 1800, ms) leads to an increase in the peak current in the test pulse (maximum of 27%, with *k*_super_=2.7 s^−1^, in this example). The interval between pulses was kept constant at 200 ms. (**g**) Summary of suprarecovery for progressive lengthening of the conditioning pulse for GluA2 WT+Stargazin (red diamonds, *n*=6–12) and GluA2 WT (empty grey diamonds, *n*=6–8) at an interval of 200 ms. Error bars represent s.e.m.

Our model also predicted unexpected properties of AMPAR-Stargazin complexes, which we could confirm experimentally. First, the model predicted a surprising overshoot in the recovery from desensitization curve for mutants with less stable desensitized states. The model predicted transient accumulation of complexes with higher conductance open states during long glutamate exposures, intended to desensitize receptors for measurements of recovery from desensitization. Thus, the response to the second pulse could in principle exceed that of the initial peak, producing a hump in the recovery curve if receptors recover before this potentiating effect dissipates. In receptors with slower recovery like GluA1, this effect should simply make recovery from desensitization apparently faster (Fig. 4a,b), in line with previous studies reporting an increase in the rate of recovery from desensitization induced by Stargazin^7^,^20^. In light of the model prediction, we hypothesized that the faster recovery previously observed in the presence of Stargazin could be an artefact of increased activity of the complex at the end of long conditioning pulses used to drive the receptor into the desensitized state in recovery protocols, rather than a true increase in the rate of recovery. Indeed, traces recorded from GluA2 WT and mutants with fast recovery, such as GluA2 R675S, showed that the current recovered to a higher level than the first peak and then decreased to the initial level. As predicted by the mechanism (Fig. 4b), this overshoot was bigger for fast recovering mutants (105±0.7%, *n*=25 for GluA2 WT and 118±3%, *n*=7, for GluA2 R675S, peak overshoot reached between 190 and 390 ms (Fig. 4c,d), because they are more strongly modulated by Stargazin, and recover to full-amplitude much faster than the boost to gating dissipates (Fig. 4b,d). This biphasic behaviour was not detectable for mutants with slower recovery kinetics, and thus less-available open states, which showed no change in the rate of recovery when coexpressed with Stargazin (Fig. 4b and Supplementary Table 1). Although we could not assess relief of polyamine block in individual channels, no change in the peak current *G*–*V* relation was detected during the recovery phase (Supplementary Fig. 5A), suggesting activity-dependent relief of polyamine block (unlikely at +50 mV)^21^, was limited. A conventional monoexponential fit suggested faster recovery in the presence of Stargazin, as reported in earlier studies^7^. However, when the data were more appropriately fitted using the sum of two exponentials, including a decaying phase corresponding to the dissipation of the potentiation from the long pulse, no significant difference in the rate of recovery from desensitization was observed between GluA2 receptors expressed with Stargazin and GluA2 receptors alone (Fig. 4e). These results indicate that Stargazin does not affect the rate of exit of the receptor from the desensitized state much. As predicted by the model, conditioning pulses longer than those we normally use led to a progressively bigger accumulation (at a rate of ∼2.8±1 s^−1^) of high-activity receptors, thus producing an even more profound overshoot (15±2%, *n*=12 Fig. 4f,g and Supplementary Fig. 5B,C). The effect, that we term suprarecovery, can be explained by an increase in the activity of the receptor-Stargazin complex in an open state with a higher mean conductance. Strikingly, without being constrained on these data, the mechanism in Fig. 3a predicted that suprarecovery should develop at a rate of about 3 s^−1^ and to about the same extent (Supplementary Fig. 5D).

### The slow-augmenting current corresponds to superactivation

**Figure 5:**
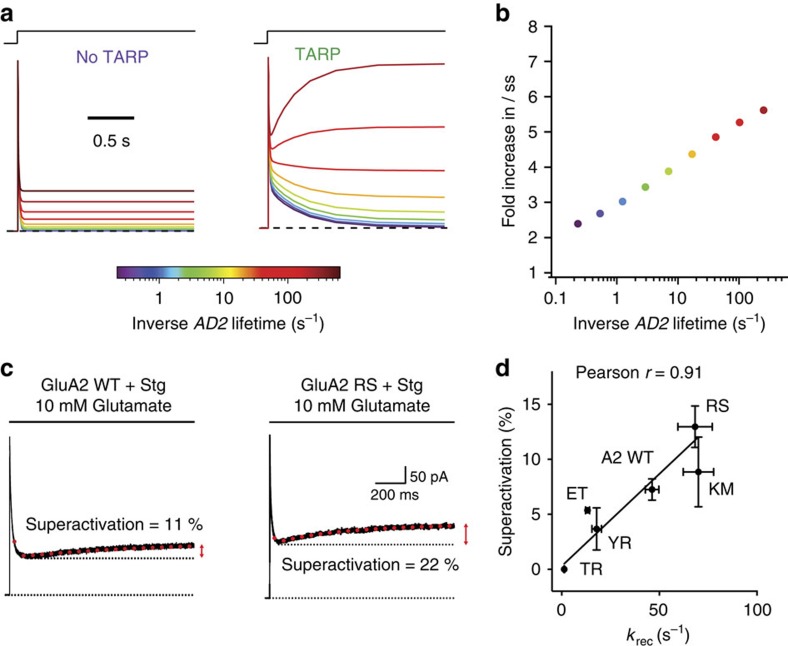
Superactivation of AMPA receptors by TARPs. (**a**) The TARP model predicted slow augmentation of current during long (>1 s) glutamate pulses. (**b**) Predicted correlation between recovery rate and fold-increase in steady-state current with TARPs (see Fig. 2b). (**c**) Long application (5 s) of glutamate to GluA2 WT coexpressed with Stargazin induced a slowly increasing current (11%, *k*=1.8 s^−1^, left), which was larger for faster-recovering mutants (GluA2 R675S, 22%, *k*=1.5 s^−1^, right) (**d**) Correlation between superactivation and recovery rate (Pearson *r*=0.91, 95% confidence interval 0.38–0.99, *n*=6–34). Error bars represent s.e.m.

The model that we propose predicts that in the continuous presence of glutamate, receptors with a less stable desensitized state (AD2) should show a progressive accumulation in the high conductance state ARS* (Fig. 5a). Critically, the mechanism predicted the linear correlation between recovery rate and fold-increase in steady-state current induced by Stargazin (Figs 2b and 5b). This correlation occurs because glutamate efficacy is decreased for mutants with faster recovery. According to the mechanism we propose, this increases the disparity between basal gating and channel gating when Stargazin is activated. Ensuring that detailed balance is obeyed requires mutants with lower efficacy shift the equilibrium towards activated Stargazin (the balance between the transitions s*+ and s*–) when the channel is open.

Indeed, fast recovering receptors such as GluA2 R675S showed a slow augmentation of the steady-state current during a 5 s application of glutamate when coexpressed with Stargazin (increasing by 13±2% with rate 1.6±0.3 s^−1^, *n*=10; Fig. 5c). This increase in the steady-state current is reminiscent of ‘resensitization', previously observed with γ-4, γ-7 and γ-8 (refs 22, 23). In contrast to these studies, but as predicted by our model, we could observe a slowly augmenting current for GluA2 WT-Stargazin complexes during long application of agonist (increasing by 8±1% with rate 1.5±0.2 s^−1^, *n*=24; Fig. 5c). The increase in the steady-state current is essentially absent for receptors with comparatively stable desensitized states, leading to a further strong correlation, between superactivation and the rate of recovery from the desensitized state of the receptor, as expected for our model (*R*^2^=0.84; Fig. 3d and Supplementary Fig. 6B). On the other hand, there was no correlation of superactivation extent to the magnitude of the current, a surrogate of receptor density (Supplementary Fig. 6C,D), providing further evidence against any effects based on association of TARPs into complexes. Finally, we back-extrapolated the overshoot during suprarecovery (for a 400 ms pulse) and found it was very similar in magnitude (13±2% *n*=26) to the superactive current, providing further evidence that the same mechanism underlies both superactivation and the suprarecovery.

Despite the evident predictive power of the model, we investigated alternate model geometries, mostly based on the original model to describe the mutant series^17^. Models with different geometries, rate constants and representations of the mutant series were tested in more than 300 simulations. We assessed the ability of a given model to reproduce the following key features of our data and previously published work: two components in deactivation decay; correlation between steady-state current increase and recovery rate; larger steady-state current and *P*_open_; and a left-shift in the steady-state concentration response curve^7^,^8^. Of these, the correlation between steady-state current increase and recovery rate was the most sensitive metric, because almost all model geometries and combinations of rate constants failed to reproduce this phenomenon.

A key alternate hypothesis for the mechanism of Stargazin modulation is that Stargazin increases activity by slowing glutamate unbinding^13^. However, at the level of a simplified model (Supplementary Fig. 7), including a higher conductance open state, such a scheme failed several of the biophysical criteria outlined above. First, entry to desensitization was not slowed by the additional TARP states (Supplementary Fig. 7B), because in saturating agonist, the desensitization relaxation is not contingent on the unbinding rate of glutamate. Secondly, such a model only predicted a hump in recovery curve because of TARPs (Fig. 4) when recovery was so fast that receptors no longer desensitized at all (Supplementary Fig. 7F). Third, recovery from desensitization depends on glutamate dissociation, with more potent ligands slowing recovery substantially^24^. Unsurprisingly, in a model where dissociation is slower from the TARP-active state (Supplementary Fig. 7F), overall recovery can only be slower, whereas in experimental observations with Stargazin it is always apparently faster^7^ (Fig. 4) or unchanged (see Supplementary Table 1 online). Fourth, and most critically, a model in which binding principally changed (compare Figs 2b and 5b and Supplementary Fig. 7G) did not reproduce the positive correlation between the increase in steady-state current and the recovery rate for our mutant series. We therefore concluded that, on the level of simple models at least, substantial changes in gating are much better at reproducing the observations than changes in binding. We cannot rule out much more complicated combinations of changes induced by Stargazin, but they are not necessary to describe the observed experimental data.

### Removing desensitization boosts superactivation

**Figure 6:**
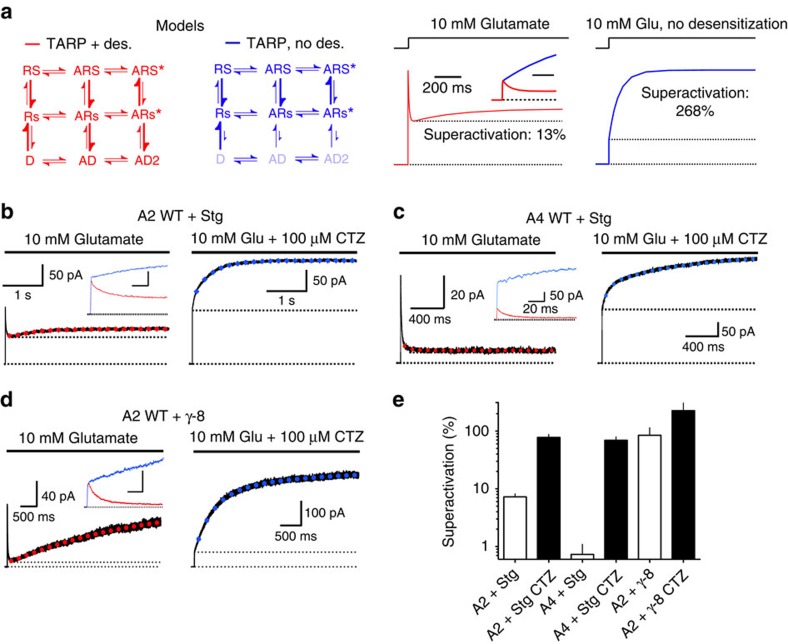
Blocking desensitization enhances TARP-induced superactivation. (**a**) Removing desensitization from the model (blue traces and scheme, see Methods) predicted that long glutamate applications should exhibit greatly enhanced superactivation, compared with the model with desensitization intact (red). (**b**) Representative traces from GluA2 WT with Stargazin evoked by 5 s application of glutamate in the absence (*k*_super_=1.7 s^−1^, left) and presence (*k*_super_=2.5 s^−1^, right) of CTZ. Abolishing desensitization greatly enhanced superactivation (from 4 to 85% for this trace). (**c**) When desensitization was intact, increases in the steady-state current for GluA4-Stargazin complexes in response to long application of 10 mM glutamate were barely detectable. Pre-exposure of the patch to cyclothiazide (CTZ, 100 μM) unmasked superactivation (from 0.2%, left panel, to 97%, right panel). Traces pre- and post-incubation with CTZ are overlaid in the inset. (**d**) Superactivation by γ-8 was also enhanced by CTZ (from 150 to 450% in this example, *k*_super_*=*1.7 and 1.8 s^−1^, in the absence and presence of CTZ, respectively). (**e**) Bar graph summarising superactivation in the presence and absence of CTZ for GluA2+Stargazin (*n*=24 and 4), GluA4+Stargazin (*n*=3) and GluA2+γ-8 (*n*=8 and 11). Error bars represent s.e.m.

A report that removal of desensitization abolishes the slowly augmenting current concluded that TARPs can reverse desensitization^23^. Cyclothiazide (CTZ) blocks desensitization, but does not induce slow augmentation of TARP-less receptor currents during long applications of saturating agonist^25^,^26^. In contrast, our mechanism predicts that removing desensitization should make the TARP-induced increase in the steady-state current during long glutamate exposures much more profound, because desensitization otherwise acts as a sink, reducing the fraction of active receptors (Fig. 6a). In the absence of desensitization, our mechanism predicted that all receptors are in principle available to demonstrate the boost to gating due to Stargazin (Fig. 6a). As predicted, the GluA2 WT-Stargazin complexes showed a much larger slowly augmenting current when preincubated in CTZ (increasing by 60, ±10%, *n*=4; Fig. 6b,e). The same phenomenon was also revealed for GluA4 WT by CTZ (Fig. 6c,e). These data indicate that the slow increase of the steady-state current is not related to the reversal of desensitization, but rather to occupancy of open states. We thus term this mechanism ‘superactivation', because complexes become progressively more active. Consistent with the stronger effects of the TARP γ-8 on AMPA receptor gating^12^, currents from cotransfection of GluA2 WT with γ-8 showed massive superactivation in the presence of CTZ (220±60% with CTZ, compared with 50±8% when desensitization is intact, *n*=11 and 8, respectively; Fig. 6d,e). With γ-8, we could also detect superactivation at negative potentials, both in the presence and absence of CTZ. The background, polyamine-sensitive current was on average 20-times larger at negative potential (Supplementary Fig. 8A). However, the magnitude of superactivation was about twice as large at –60 mV, in line with expected relief of polyamine block (Supplementary Fig. 8B,E). Magnitude effects are subject to rundown and other artefacts but critically, the rate of superactivation was voltage independent for GluA2-γ-8 cotransfections (Supplementary Fig. 8D). This result provides further evidence that voltage effects on AMPAR gating are minor compared with superactivation, which is robust at both positive and negative potentials. TARP-induced superactivation was probably previously missed in some experiments because some TARPs like γ-8 either express poorly or are bad at incorporating into complexes. Thus modulatory effects were overwhelmed in a mixed population by receptors with sub-stoichiometric TARP content. Our data suggest that superactivation is a general property shared by all TARPs and it is not AMPAR-subunit dependent, but merely a thermodynamic consequence of the boosted gating that all TARPs promote. Given the widely held view that AMPAR gating is modified by TARPs at synapses^11^, and that desensitization is not a prerequisite, superactivation is likely to occur *in vivo*.

### Superactivation of trains of pulses

**Figure 7:**
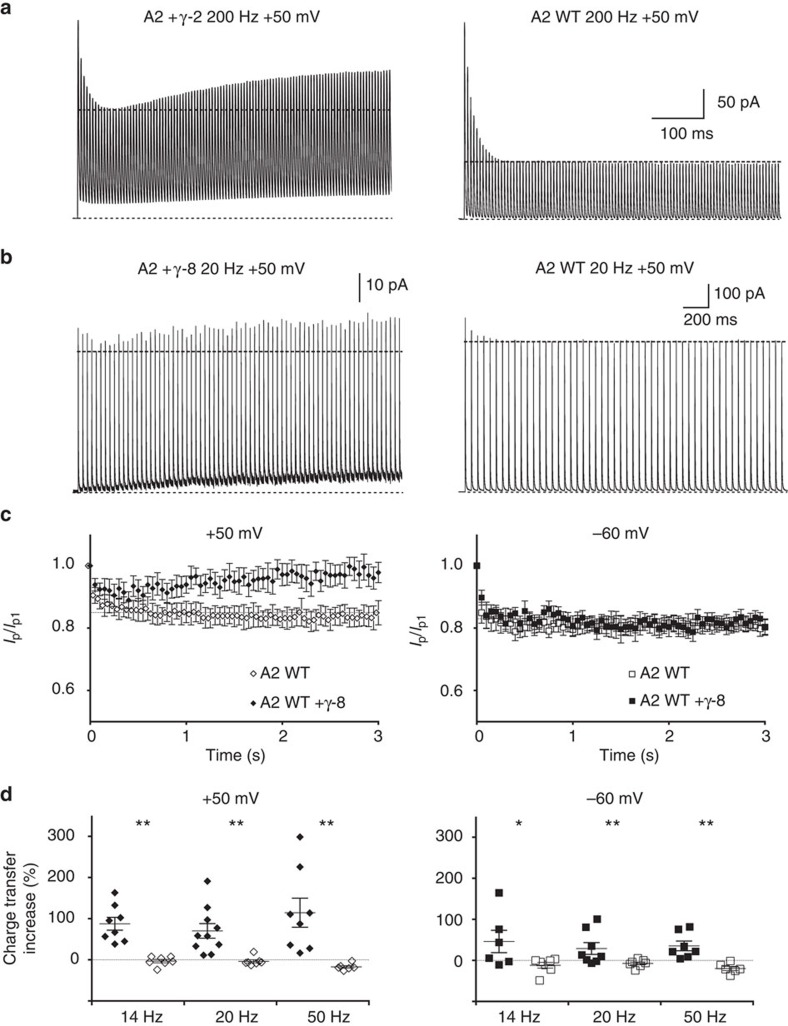
Train stimulation induces superactivation. (**a**) 200 Hz trains of 1 ms pulses of 10 mM glutamate, to mimic intense synaptic activity, induced superactivation of currents from GluA2-Stargazin complexes (in this example, 23%). (**b**) GluA2 WT receptors coexpressed with γ-8 showed superactivation in response to lower frequency stimulation (20 Hz; steady-state potentiation in this example was 31%). (**c**) Summary of peak current, normalized to the first peak in the train, during 20 Hz train stimulation for A2+γ-8 and A2 alone at +50 (left panel; *n*=8 and 4, respectively) and −60 mV (right panel; *n*=7 and 4, respectively). (**d**) Summary of charge transfer increase during train stimulation for A2+γ-8 and A2 alone at +50 (left panel; *n*=8–10 and 6–7, respectively) and −60 mV (right panel; *n*=8–10 and 6–7, respectively) recorded at different frequencies. The charge transfer increase was calculated as the ratio between the change in the charge transfer during the last five pulse of the train and the change during the first five pulses (see Methods). A nonparametric randomization test was used; *P<0.05, **P<0.01.

To assess if superactivation occurs in a more physiological situation, we next stimulated patches with trains of 1 ms pulses, mimicking intense synaptic activity. Such trains depressed responses of wild-type and mutant receptors, as predicted by our models (Supplementary Fig. 9A,B). However, following initial depression, peak currents for GluA2 WT receptors coexpressed with Stargazin subsequently recovered during the train to a higher response that could be maintained indefinitely (peak currents increased by 10±1% and 8±1% compared with the extent of the greatest depression, at 200 and 100 Hz, *n*=24 and 13, respectively; Fig. 7a). As predicted by the mechanism, complexes incorporating the fast recovering mutant GluA2 R675S and Stargazin exhibited more profound superactivation during trains (14±4%, *n*=4 at 200 Hz; Supplementary Fig. 9B). In line with the greater propensity of γ-8 to potentiate AMPAR gating, GluA2-γ-8 complexes showed robust superactivation during lower frequency trains, from 50 to 5 Hz (50±0.4% *n*=2, 20±0.1% *n*=2, 20±0.9% *n*=4 and 8±0.4% *n*=2 at 50, 20, 14 and 5 Hz respectively; Fig. 7b,c). The increased activity of the channel during train stimulation was evident also from the increase in the charge transfer at the end of the stimulation compared with the beginning (90±15 and −3±4% at 14 Hz, *P*=0.0006, *n*=8 and 7; 150±80 and −4±4% at 20 Hz, *P*=0.002, *n*=10 and 7; 120±35 and −15 ±3% at 50 Hz, *P*=0.0003, *n*=8 and 6, for GluA2+γ-8 and GluA2 WT, respectively; Fig. 7d). This effect was due to the lengthening of the current decay and increase of the standing current (Supplementary Fig. 10), more than any potentiation of amplitude. Consistent with the expected larger contribution of superactivation at negative voltage (Supplementary Fig. 8E), increased charge transfer was evident also at negative potentials, even though the majority of receptors were not heavily TARPed enough to be relieved of polyamine block (60±30 and −15 ±7% at 14 Hz, *P*=0.01, *n*=6 and 8; 30±15 and −7±4% at 20 Hz, *P*=0.02, *n*=8 and 7; 40±10 and −20±5% at 50 Hz, *P*=0.003, *n*=7 and 6, for GluA2+γ-8 and GluA2 WT, respectively; Fig. 7d). Unsurprisingly, wild-type GluA2 without γ-8 exhibited only depression of amplitude and charge transfer (Fig. 7c,d).

Although our mechanism predicted superactivation and other previously unheralded properties of TARPed AMPA receptors, we were concerned that these phenomena might also represent the association of Stargazin into sub-stoichiometric complexes^15^. The relief of polyamine block, being separable from kinetic effects (Supplementary Fig. 3C), allowed us to assess the average TARP content of the complexes. Relief of polyamine block in cells coexpressing GluA2 and Stargazin was less profound than for the GluA2-Stargazin tandem (Fig. 1b), presumably because some complexes contain less than four Stargazin molecules. If Stargazin were recruited into these complexes during prolonged or repetitive receptor activation, we would expect less block following stimulation that induced superactivation. Measurement of the *G*–*V* relations, before and after such trains, failed to reveal differences in polyamine block of similar magnitude to those between coexpression and tandem (Supplementary Fig. 11). At the peak of suprarecovery, similar results were obtained (Supplementary Fig. 5). Further, superactivation was rapidly inducible and reversible, recurring on a trace-by-trace basis, and had no dependence on the apparent density of channels (Supplementary Fig. 6). These findings suggest that superactivation is an intrinsic property of the receptor-TARP complex, and not the result of decreased polyamine block and/or recruitment of TARPs into complexes.

## Discussion

Here, we propose that Stargazin exists in two states: a basal state that only reduces polyamine block and an active form that is promoted by receptor opening, and which additionally increases both channel conductance and glutamate efficacy. We establish that this simple principle, which allowed us to formulate the first consistent model for the activation of AMPAR-TARP complexes, has major implications for synaptic transmission. Evidence is emerging that TARPs promote slow gating modes^11^,^23^,^27^ and our data demonstrate that switching into the high-activity mode is driven by receptor activity, perhaps subject to regulation by other auxiliary subunits^23^. In contrast to most previously published work, we examined pure populations of AMPA receptors in complex with TARPs, and thus were able to ensure that these distinct levels of activity arise from complexes. The unexpected properties of complexes that we isolated were missed in previous experiments, probably because these experiments examined mixtures, which tended to mask the effects of TARPs. Published effects on mixtures also likely suffered from variability because they were subject to the variable expression levels between individual cells.

The model we built depended on our observation that relief of polyamine block and the kinetic effects of Stargazin are separable, perhaps because they result from physically distinct interactions. Our model is agnostic to the sites of interaction between AMPARs and TARPs, but indicates that conformational changes alone, and particularly those involved in channel gating, rather than any change in glutamate affinity (Supplementary Fig. 7), are sufficient to describe non-equilibrium behaviour of complexes. Importantly, even though the mutations we used are within the AMPAR ligand-binding domain, their effects on average activity in saturating glutamate are the most important factor in their propensity to alter the effects of Stargazin, rather than any direct interaction. Notably, this factor may also explain the GluA-subunit specificity of modulation^20^. On the other hand, the effects on polyamine block seem likely to arise from membrane interactions^28^ that are present consistently when complexes include TARPs, irrespective of mutant kinetics.

The idea that Stargazin has two states explains the two components in the deactivation decay, as well as the activity-dependent accumulation of superactive receptors in various situations. Although other mechanisms producing both short and long bursts of openings could explain these two components, single-channel recordings of GluA4 coexpressed with TARPs reveal that, in addition to long bursts, some channels in the patch display brief activations just like receptors without TARPs^8^,^19^. This property is explicit in the formulation of our model, because a layer of states with much higher efficacy of opening supplements the original model without TARPs. AMPA-type glutamate receptor-TARP tandem constructs also display two classes of activity: brief bursts like TARP-less channels and very long high conductance bursts^19^, which could correspond to the two distinct open–closed reactions in our model. Although in principle, we propose that the complex has two states overall, for the open channel to have different stabilities in the presence of TARPs^8^,^19^, distinct interactions between TARPs and the receptor are required in each condition. We cannot exclude the remote likelihood that this occurs without any conformational change at all in the TARP, but it appears far more plausible that, just as the receptor can change conformation between states driven by ligand-binding, TARPs can change conformation, promoted by gating of the receptor, to stabilize a superactive form of the complex.

Despite its obvious physical disconnection from the multiple subunits of the tetramer, the model we propose has a strong predictive power and shows the degree to which surprisingly complex kinetic behaviour can derive from a fairly simple scheme. While more complex and physically accurate models were likely to provide more exact descriptions (see below), we sought to find a simple model that could describe existing data and generate robust predictions that we could test. To what extent can such a simplistic model be expected to describe AMPA receptor gating? The single binding site can be thought to represent one subunit because there is no strong cooperativity of neurotransmitter binding in AMPA receptors^29^. We included a high-efficacy open state with 2.5-fold higher conductance to describe the generally higher conductance of long bursts in single-channel recordings^8^,^19^. Notably, models that had only a high-efficacy state, with the same average conductance as the regular open state, predicted that suprarecovery would be barely detectable, and absent in wild-type channels (Supplementary Fig. 12). However, the size of superactivating currents during long glutamate applications placed an upper limit on this increased conductance.

The model we propose has some limitations in describing glutamate receptor activation. Our model does not predict autoinactivation^16^,^30^, perhaps because we only considered complexes, and autoinactivation might be due to TARP dissociation. Also, our original scheme cannot replicate the very rapid dissipation of current for the slow recovering mutants during trains, (Supplementary Fig. 9) unless desensitization rates are faster than observed during long pulses. These models with one binding site cannot capture the subtleties of partially liganded channels or sublevels, which we suspect are responsible for these deficits. Such deficits do not detract from the predictive power of our TARP model with respect to superactivation.

Positive feedback loops are essential for numerous physiological processes, including the Ferguson reflex that initiates childbirth^31^ and locomotion^32^. However, such feedback usually encompasses loops including transcription, post-translational modifications of multiple cellular components^33^, or networks of cells. In contrast, activity-induced superactivation of AMPAR-TARP complexes is a much more rapid positive feedback mechanism, entirely contained within a macromolecular complex. We are unaware of reports of similar mechanisms within protein complexes, but the fast onset (within a second) is necessary for the complex to be able to respond fast enough to have consequences for receptor activity at synapses. TARPs alter the gating of synaptic AMPA receptors, producing slow deactivating synaptic currents^11^,^34^. According to our mechanism, it is the small fraction of receptors that are basally superactive that lengthens the decay of individual synaptic potentials. The kinetics of AMPARs can contribute to short-term synaptic plasticity. Receptor desensitization permits short-term depression at some synapses^35^,^36^,^37^. On the other hand, facilitation is exclusively attributed to increased release probability following presynaptic calcium accumulation, possibly because postsynaptic mechanisms for short-term potentiation are lacking. This deficit is notable in the context of target cell-specific short-term potentiation, to date ascribed to retrograde signalling to the presynaptic terminal^38^. Superactivation of AMPAR-TARP complexes is a positive feedback mechanism that could in principle generate short-term potentiation at a purely postsynaptic locus. We envisage this effect would develop during repetitive activity within hundreds of milliseconds (Fig. 7), and dissipate on the timescale of ∼1 s (Fig. 4). The expression patterns of TARPs with differential power to modulate AMPA receptor gating (for example, Stargazin versus γ-8) might balance superactivation against the frequency of synaptic inputs according to brain region, or to afford cell specificity.

## Methods

### Molecular biology

We used GluA2 and GluA4 flip receptors, unedited at the pore site (Q-containing) in the pRK vector also expressing eGFP following an internal ribosomal entry site sequence. Mouse Stargazin was a kind gift from Susumu Tomita and was subcloned by PCR into the pRK8 vector containing the non-cytotoxic version of dsRed (dsRed-Max, Addgene plasmid 21718)^39^ also under the control of an internal ribosomal entry site. Mouse γ-8 (a kind gift from Roger Nicoll) was subcloned into the same vector. To construct the GluA2-Stargazin tandem, the C-terminus of GluA2 was directly fused to the N-terminus of Stargazin (as previously done with GluA1)^16^. The unique restriction sites EcoRI and XbaI were inserted by PCR at the 5′ and 3′ of the GluA2 coding sequence and the stop codon eliminated. Point mutations were introduced by overlap PCR and confirmed by double-stranded sequencing. Residues were numbered based on the assumption that the signal peptide of GluA2 is 21 residues.

### Cell culture and electrophysiology

HEK-293 cells (DSMZ, Germany) were transfected with a total of 3 μg DNA using calcium phosphate. Wild-type and mutant GluA receptors were cotransfected with Stargazin or γ8 at a ratio of 1:2 and 1:5, respectively. Cells transfected with Stargazin were supplemented with 30 μM NBQX (Abcam, UK). Cells were recorded 24–48 h after transfection at room temperature. Outside out patches were voltage-clamped at a holding potential of either –60 or +50 mV for kinetic measurements. Currents were sampled at 20 kHz and low-pass filtered at 10kHz using an Axopatch 200B amplifier (Molecular Devices, USA) and acquired with Axograph X software (Axograph Scientific, USA). The external solution contained: 150 mM NaCl, 0.1 mM MgCl_2_, 0.1 mM CaCl_2_ and 5 mM HEPES, titrated to pH 7.3 with NaOH, to which we added drugs as required. The pipette solution contained: 120 mM NaCl, 10 mM NaF, 0.5 mM CaCl_2_, 5 mM Na_4_BAPTA, 5 mM HEPES and 0.05 mM spermine, pH 7.3. Chemicals were obtained from Carl Roth International (Germany). We applied ligands to outside out patches via a piezo-driven fast perfusion system (PI, Germany). In the experiments in which CTZ (100 μM; Hello Bio, UK) was used to reduce receptor desensitization, we included it in both the wash and glutamate barrels of the perfusion tool. Typical 10–90% solution exchange times were faster than 300 μs, as measured from junction potentials at the open tip of the patch pipette.

### Data analysis

Receptor desensitization and deactivation were measured by applying 10 mM glutamate for 500 and 1 ms, respectively. Both desensitization and deactivation rates were calculated using a two exponential fitting. Rates constants are expressed as weighted mean of multiple components. To measure recovery from desensitization, we used a two-pulse protocol with a variable inter pulse interval. Recovery data were fitted by a Hodgkin–Huxley-type function:


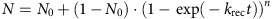


where *N* is the active fraction of receptors at time *t* following the first pulse, *N*_0_ is the active fraction at the end of the conditioning pulse and *k*_rec_ is the rate of recovery. Recovery data from receptors showing suprarecovery were fitted using the sum of two exponentials:


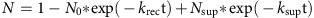


where *N* is the active fraction of receptors, *N*_sup_ is the amplitude of superactivation and *k*_sup_ is the rate of dissipation of superactivation.

To measure superactivation, we applied glutamate for 5 s and fitted the decaying and slowly augmenting currents with a triple exponential function (without CTZ) or the slowly rising current with a one- or two-component exponential (with CTZ). Where appropriate, multiple components were combined into weighted time constants for comparisons. Superactivation was defined as the excess steady-state amplitude over the trough of the current following the initial peak, normalized to the peak current. This definition is in principle the same as for resensitization as previously published, and so values can be directly compared^23^. For measurements with CTZ, the initial fast peak corresponding to glutamate activation (within 1–2 ms of glutamate application beginning) was taken as the baseline for superactivation. For measurements of responses paired according to positive and negative voltage, we excluded one patch where the rundown was so severe that the initial fast peak was similar in size at negative and positive potential. The charge transfer during train stimulation was determined by integrating the current trace over time. The change in the charge transfer (Δ*Q*) was defined as follows:


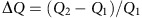


where *Q*_1_ was the charge transfer measured during the first pulses of the train and *Q*_2_ was the charge transfer measured during the last five pulses.

At least 5–6 patches from at least three different tranfections were obtained for each condition, whenever possible. In case of more challenging experiments, at least three patches were recorded. No data were excluded, except from patches where recordings were unstable, had excessive rundown or solutions exchange was slower than 0.5 ms. Results are presented as mean±s.e.m. Statistical significance was assessed with nonparametric test, to avoid any unwarranted assumptions about the distribution of the data. For most comparisons we made a two-tailed, unpaired Randomization test, with 5,000 to 1 × 10^6^ runs, using the program RANTEST, available at https://code.google.com/p/dc-pyps/. ANOVA and linear regression was done in Graphpad PRISM.

### Kinetic modelling

Modelling was done using home-written routines^17^ incorporated in the PYTHON package Aligator (Analysis of Ligand Gating: trains and other relaxations; the collection of scripts is available from github.com/aplested/aligator). The rates were adjusted by hand, paying attention to reproduce macroscopic rates for wild-type GluA2 including entry to desensitization (∼100 s^−1^), deactivation (1,000 s^−1^) and recovery from desensitization (∼20 s^−1^). The adequacy of particular geometries of rate alterations that maintain microscopic reversibility is not necessarily detectable in equilibrium measurements, such as concentration response curves, but is revealed by relaxations. For example, the relaxation rates from Stargazin active states is determined by the relaxation from superactivation in recovery protocols, and the activation rates of Stargazin (for example, s*+) was tightly constrained by the development of the slow-augmenting current during long applications of glutamate. Altering other rate constants than *d*2–, *d*2*–, *α* and s*+ generally failed to describe previously published observations, or the correlation between increase in steady-state current with Stargazin and the recovery rate for our mutant series.

In the final simulations based on the model in Fig. 3b, the rate constants (per second), before adjusting for microscopic reversibility in each trial, were as follows: s*+=15; s*–=3; *s*+=0.07; *s*–=1; *β*=8,000; *α*=3,000; βs=500,000; *d*2*+=120; *d*2*–=2; *d*2+=120; *d*2–=5; *d*1+=300; *d*1–=25; *d*0+=1; *d*0–=3; *k*+=5,000,000; *k*–=40,000; and *k*d–=2,500. Applicable rate constants were the same for the less-complex model without TARPs in Fig. 3a. The normalized mean conductances of the open states were: state ARs*: 0.4 and ARS*: 1 (except in Supplementary Fig. 11 where the conductances were equal). To model block of desensitization by CTZ, rates *d*0+, *d*1+ and *d*2*+ were set to 0.1 s^−1^. The kinetics of the GluA2 mutant series can be relatively well described by changing the lifetime of the deep desensitized state using the model in Fig. 3a. We thus varied the rates *d*2– and *d*2*– as indicated in Table 1.

Trial 0 represented a slow recovering mutant (for example, GluA2 E713T/Y768R) and Trial 8 a fast recovering mutant (for example, GluA2 K761M). Trial #7 gave the closest approximation to wild-type GluA2. In both mechanisms (Fig. 3a,b), these changes were compensated by modest alterations in the shutting rate of the channel (*α*), consistent with the slower deactivation in slow recovering mutants^17^. These manipulations provide a better description that that in our original report, because the deactivation decay is more accurately described. To maintain microscopic reversibility, this change was compensated in the TARP mechanism in Fig. 3b by variation in s*+, effectively changing the extent to which receptor activation promoted superactivation across the mutant series. Other changes, such varying in the TARP-active channel shutting rate separately from the basal shutting rate (*α*), or letting s*– or *s*– vary, failed to reproduce the positive correlation between increases in steady-state current induced by Stargazin and the recovery rate, or the hump in the recovery curve.

To model activation by kainate in the absence and presence of TARPs (Supplementary Fig. 4), we adjusted gating and desensitization rates from the model in Fig. 3b to mimic the weak agonism and desensitization of kainate activation of GluA2 (Q)^40^ as follows (per second): *β*=1,600; α=30,000; *d*2+=12; *d*2–=30; *d*2*–=12; and *d*1–=300. The opening rate of the superactive state (*β*s) was also reduced fivefold (that is, by the same extent as *β*) to maintain the strength of the GluA2-TARP interaction (*β*s=100,000). Other rates were the same as in the other simulations, and the corresponding rates in the absence of TARPs were held the same. The amplitude of single openings of GluA2 (Q) activated by kainate are too small to measure directly, and were previously estimated at 2.5 pS versus 21 pS for the main glutamate level^41^. In keeping with this observation, we reduced the conductance of the kainate-activated, basal open-state fivefold (normalized: 0.08), while keeping the conductance of the superactive state the same.

## Additional information

**How to cite this article:** Carbone, A. L. & Plested, A. J. R. Superactivation of AMPA receptors by auxiliary proteins. *Nat. Commun.* 7:10178 doi: 10.1038/ncomms10178 (2016).

## Supplementary Material

Supplementary Supplementary Figures 1-12 and Supplementary Tables 1-2

## Figures and Tables

**Table 1:** Rate constants used in the families of simulations shown in Fig. 3.

**Trial #**	***d*****2– (s**^−1^**)**	***d***2*– (s^−1^**)**
0	0.16	0.18
1	0.32	0.29
2	0.63	0.47
3	1.26	0.76
4	2.51	1.23
5	5.0	2.0
6	10.0	3.24
7	19.9	5.26
8	39.7	8.53
