# Supplementary material for: Superactivation of AMPA receptors by auxiliary proteins
Source: Nat Commun. 2016 Jan 8;7:10178. doi: 10.1038/ncomms10178 (PMC4729862; doi:10.1038/ncomms10178)
Supplement: Supplementary — Figures 1-12 and Supplementary Tables 1-2 [file ncomms10178-s1.pdf]

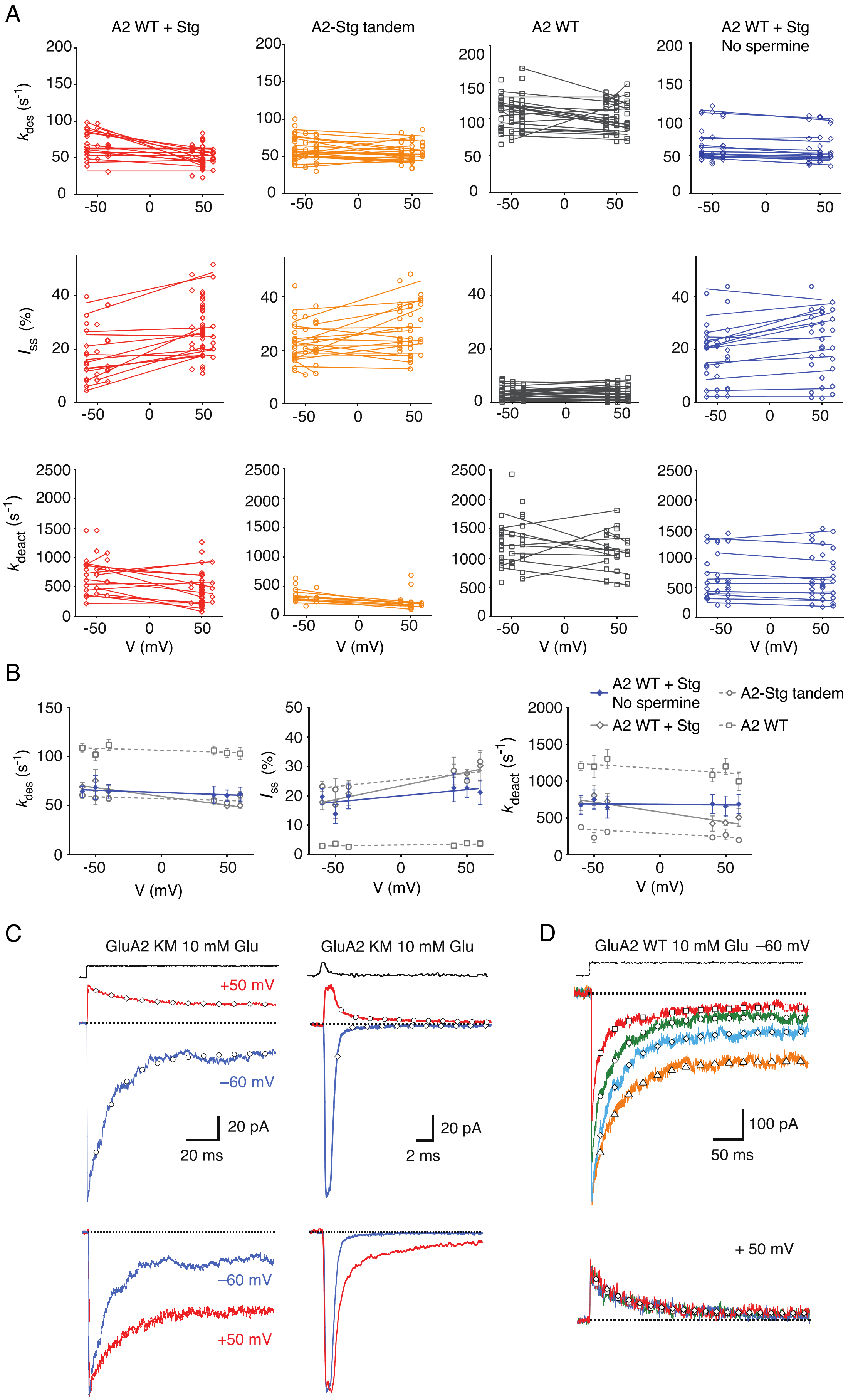

A
A2 WT + Stg
A2-Stg tandem
A2 WT
A2 WT + Stg
No spermine
k_{des} (s^{-1})
I_{ss} (%)
k_{deact} (s^{-1})
V (mV)
B
A2 WT + Stg
No spermine
A2 WT + Stg
A2-Stg tandem
A2 WT
C
GluA2 KM 10 mM Glu
GluA2 KM 10 mM Glu
+50 mV
–60 mV
20 pA
20 ms
20 pA
2 ms
–60 mV
+50 mV
D
GluA2 WT 10 mM Glu –60 mV
100 pA
50 ms
+ 50 mV

Supplementary Figure 1. Mixed populations of receptors and apparent dynamic loss of complexes. **(A)** The voltage-dependent kinetics observed in individual patches from cells cotransfected with GluA2 and Stargazin (with spermine, n = 10-44) was lost when spermine was not included in the recording pipette (blue, n = 7-14). **(B)** Fits to average data from GluA2 WT + Stargazin in the absence of spermine (blue, n = 7-14). For comparison the other three conditions are shown in grey: GluA2 + Stargazin coexpression with spermine (diamonds, n = 10-44), GluA2 alone (squares, dashed line, n = 6-23) and GluA2-Stargazin tandem (circles, dashed line, n = 4-25). Two-way ANOVA showed a very low probability of no difference between the kinetics measured -60 and + 50 mV for GluA2 + Stargazin (k_{des} p = 0.0005, k_{deact} p = 0.027; I_{ss} p = 0.0001). For the other comparisons between negative and positive voltage, $p > 0.1$ in all cases. In addition the kinetics of GluA2 + Stargazin showed a much lower probability of no difference from that of the tandem at – 60 than at + 50 mV (k_{des} p = 0.03 and 0.89; k_{deact} p = 0.04 and 0.43; I_{ss} p = 0.007 and 0.64 at – 60 and + 50 mV, respectively). Additional statistical analysis of the linear regressions, supporting our hypothesis, is shown in Supplementary Table 1. **(C)** Representative currents evoked by 10 mM glutamate in cells expressing the fast recovering mutant GluA2 K761M (KM) with Stargazin at –60 (blue) and +50 mV (red). Superimposition of the traces showed a substantially smaller apparent effect of Stargazin at negative potentials compared to positive potentials. The desensitization and deactivation rates measured at negative potentials were significantly faster than those measured at positive potential (k_{des} = 40 ± 2 and 80 ± 15, p = 0.0011, 40 and 60 s^{-1} in this example; k_{deact} = 350 ± 30 and 1740 ± 220, p = 0.00026, 150 and 2200 s^{-1}in this example, n = 16 and 6, at +50 and -60 mV respectively). Similarly, the effect of Stargazin on the steady state current was reduced at negative potentials (in this example, I_{ss} = 15 and 45 % at -60 and +50 mV, respectively). **(D)** At negative potentials, some patches showed a decrease in the effect of Stargazin on GluA2 WT with time (orange t = 0 s, blue t = 5 s, green t = 10 s and red t = 20 s). For the patch shown in the figure, the desensitization rate increased from 50 s^{-1} at t = 0 to 120 s^{-1} at t = 20 s. The latter value is the same as k_{des} for GluA2 WT alone. The same patch at positive potential showed no change in the kinetics of the normalized current, with stable k_{des} of 46 ± 1.5 s^{-1} and steady-state current of 15 ± 2 %, over an equivalent series of applications. This effect suggested that, in some patches, Stargazin containing complexes were lost over time.

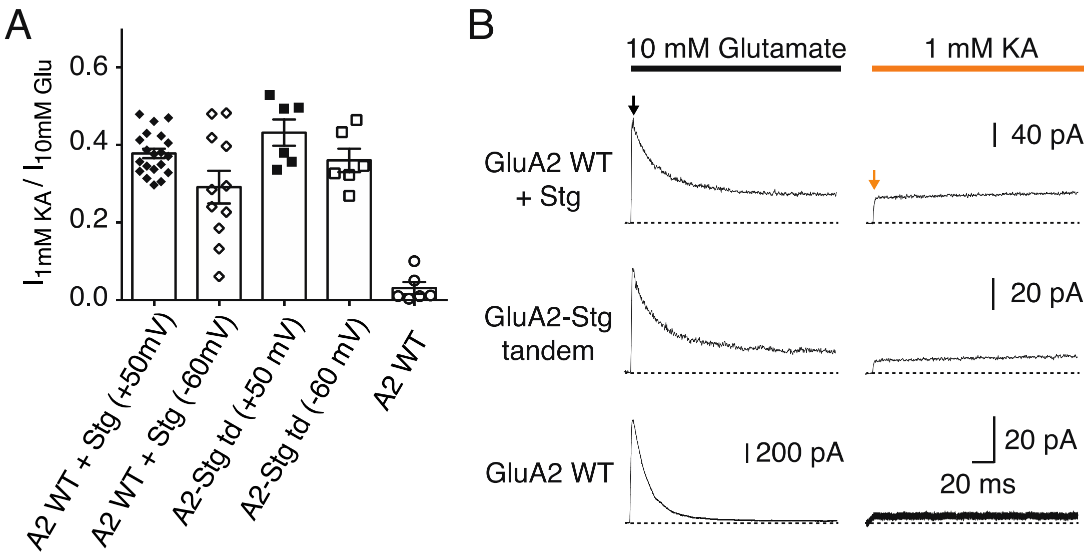

Supplementary Figure 2. Complexes isolated at +50 mV have a heavy TARP content. (**A**) Kainate (KA) efficacy at GluA2 WT coexpressed with Stargazin at +50 mV (*n* = 20), was high whereas at –60 mV (*n* = 12), the ratio was on average less and, in individual patches, spanned the range from that of the tandem dimer (*n* = 6) to the wild-type receptor without Stargazin (*n* = 6). In contrast, the KA/Glu ratio was high and voltage independent at the tandem dimer. (**B**) Representative traces recorded from cells coexpressing GluA2 WT and Stargazin, GluA2-Stargazin tandem or GluA2 WT alone in response to 10 mM glutamate (black) or 1 mM KA (orange). Arrows indicate where the current was measured. Holding potential was + 50 mV. Error bars represent s.e.m.

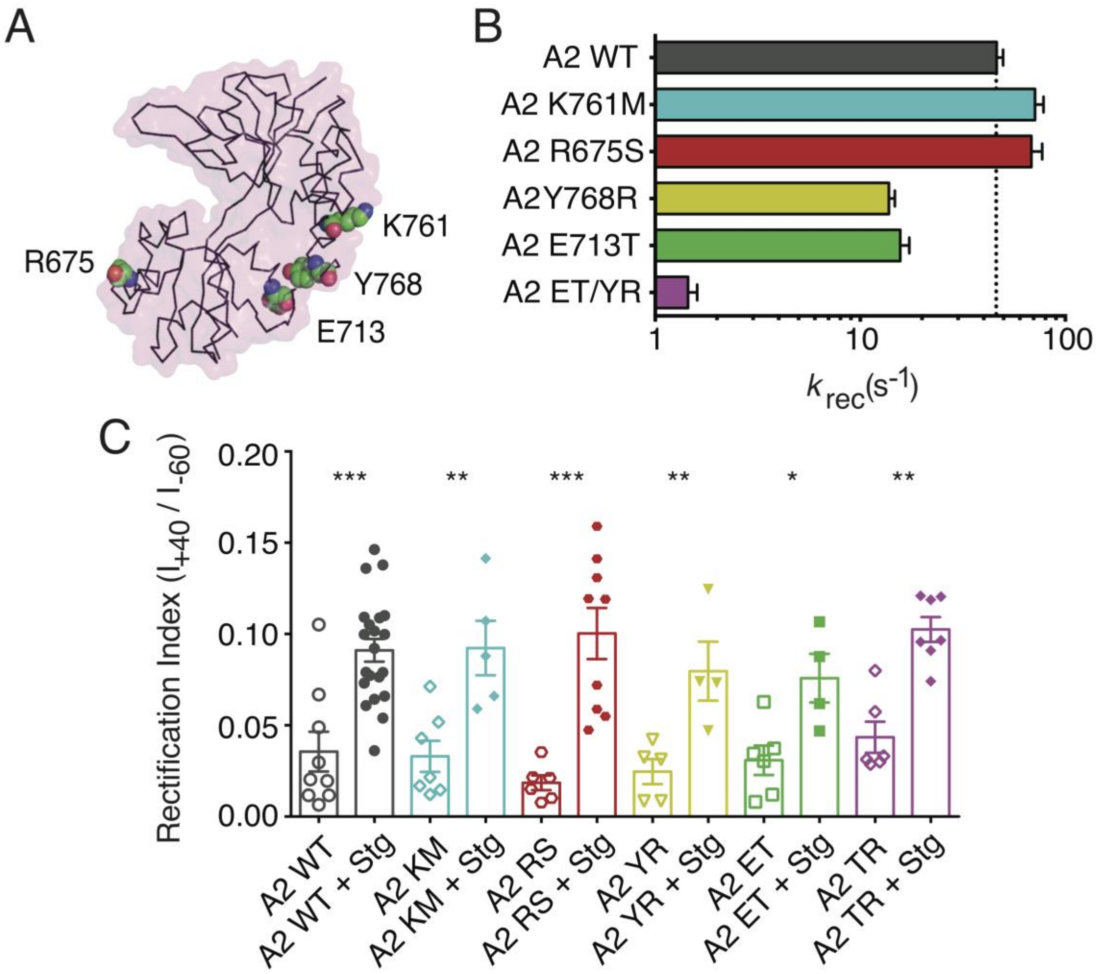

Supplementary Figure 3. Mutants of the ligand binding domain (LBD) with altered recovery properties assemble normally with Stargazin. **(A)** Cartoon showing the location of the residues mutated in the lower lobe (D2) of the LBD. **(B)** Bar graph showing the rate of recovery from desensitization of D2 mutants (data from Carbone and Plested, 2012). GluA2 R675S (RS) and K761M (KM) recover 1.5 and 1.7-fold faster than GluA2 WT, respectively. The rates of recovery of GluA2 E713T (ET) and Y768R (YR) are 2.8 and 1.8-fold slower than wild-type, respectively. Combination of GluA2 E713T and Y768R (TR) decreases the recovery rate by 25-fold compared to WT. **(C)** Rectification index (calculated as I_{+40}/I_{-60}) for GluA2 WT and mutant receptors with and without Stargazin (n = 4-17). All mutants showed robust increase in the rectification index when coexpressed with Stargazin that was highly significant, suggesting that the interaction with Stargazin is not affected in the mutant receptors (Non-parametric randomization test, * $p < 0.05$; ** $p < 0.01$; ***, $p < 0.001$, Randomization test). Colors are as in panel B. Error bars represent s.e.m.

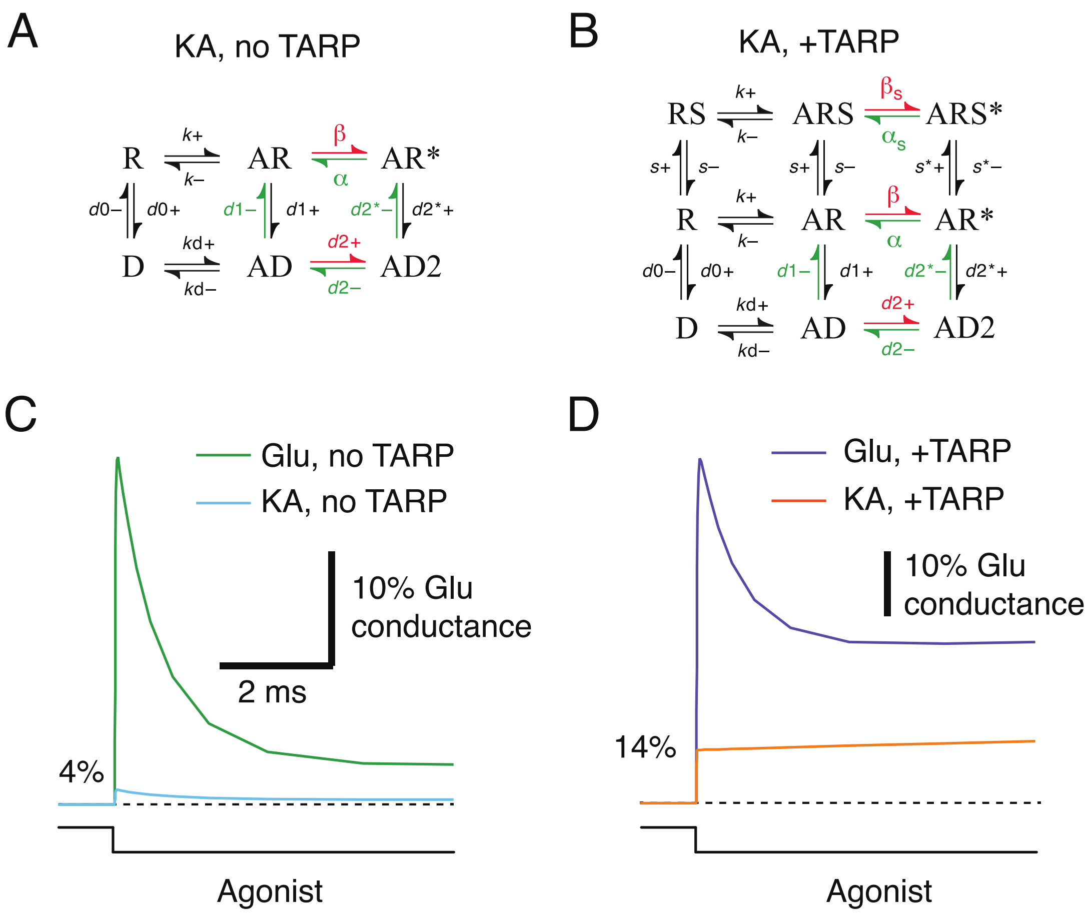

Supplementary Figure 4. Model for Stargazin-induced increase in kainate efficacy.

(**A**) Kainate was modelled as having low efficacy, and inducing weak desensitization. Rates were reduced 5-10 fold (red) or increased 5-10 fold (green); see Methods for rates used. The basal conductance (of AR*) activated by kainate was 5-fold less than that activated by Glu. (**B**) For the mechanism incorporating TARPs, we kept the same proportions of superactivation at rest and in the open state, to assume no change in receptor - TARP interaction. The conductance of the superactive open state was normalized to 1 in all simulations. (**C**) The simulation predicted a weakly-desensitizing kainate-activated current that was 4% of the amplitude of the glutamate-activated peak current. (**D**) Including TARP modulation in the model gave rise to a substantial initial current. This effect increased the KA/Glu ratio (for the initial peak currents) about 3-fold (to 14%). Note the subtle superactivation of the simulated kainate current. This effect could be discerned in some recordings (compare with Supplementary Figure 2B) and previously published work.

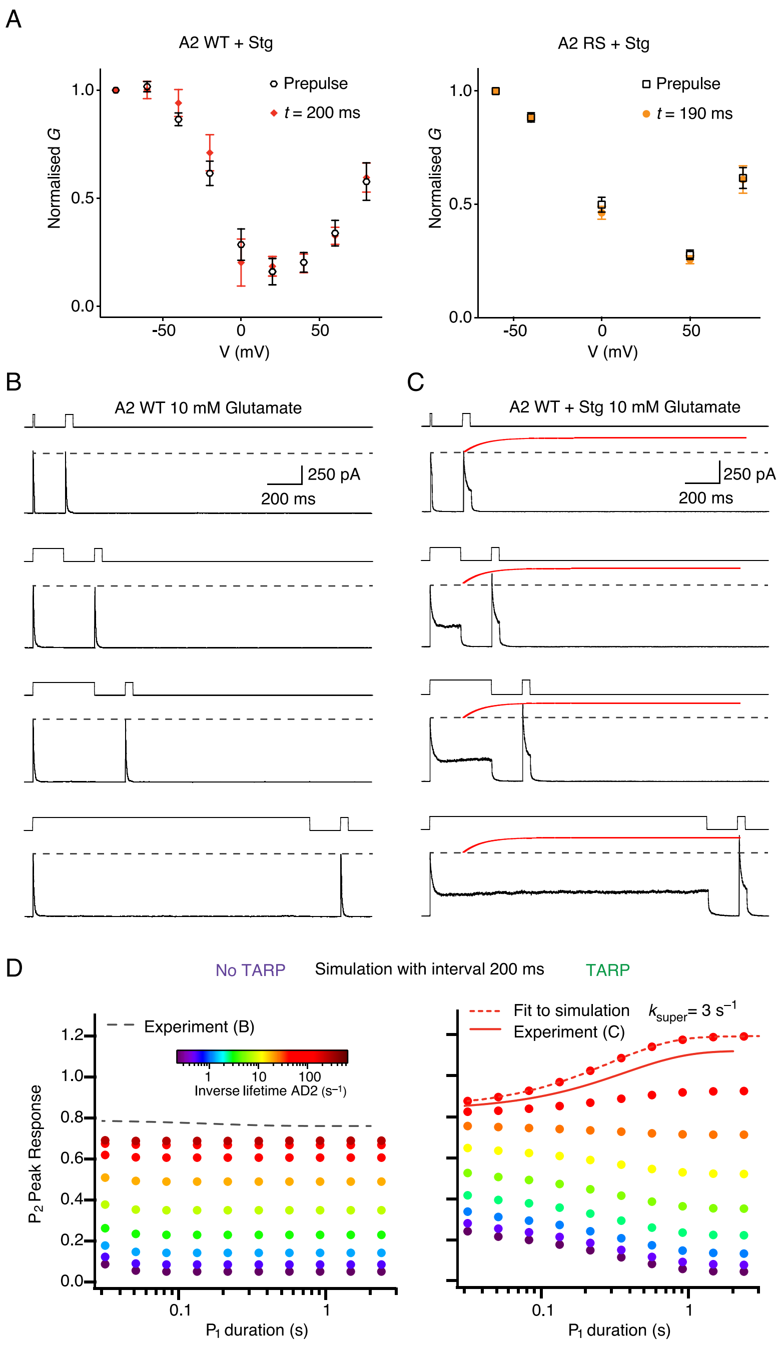

Supplementary Figure 5. Suprarecovery is not caused by relief of polyamine block but by increased receptor activity during prolonged exposure to Glutamate. **(A)** G-V relations measured before the conditioning pulse (black) and at the peak of the overshoot (red; 2nd pulse at 200 ms for GluA2 WT, *left*, n =7, and 190 ms for GluA2 R675S, *right*, n =3, both with Stargazin, see Fig. 4) showed that spermine block is unchanged during suprarecovery and thus spurious relief of polyamine block cannot account for the overshoot. **(B)** Individual traces from a patch recording of GluA2 WT alone. In the absence Stargazin, conditioning pulses of different durations have no effect on subsequent responses. **(C)** Individual traces from a patch recording of

GluA2 WT + Stargazin, showing that increasing the length of the conditioning pulse increases the amount of suprarecovery observed in the second pulse. The interval between pulses was kept constant at 200 ms. The recording is the same as shown in Fig. 4F. **(D)** Simulation using the models in Figure 3, for different lifetimes of the state AD2. The model without TARP predicts no suprarecovery, independent of the duration of the first pulse, as in panel (B). The TARP model predicts robust suprarecovery for fast recovering (red points, high inverse lifetimes of state AD2) AMPA receptors (example exponential fit, k_{super} ~ 3 s^{-1}) developing with similar rate and extent to the experiment in panel (C). Error bars represent s.e.m.

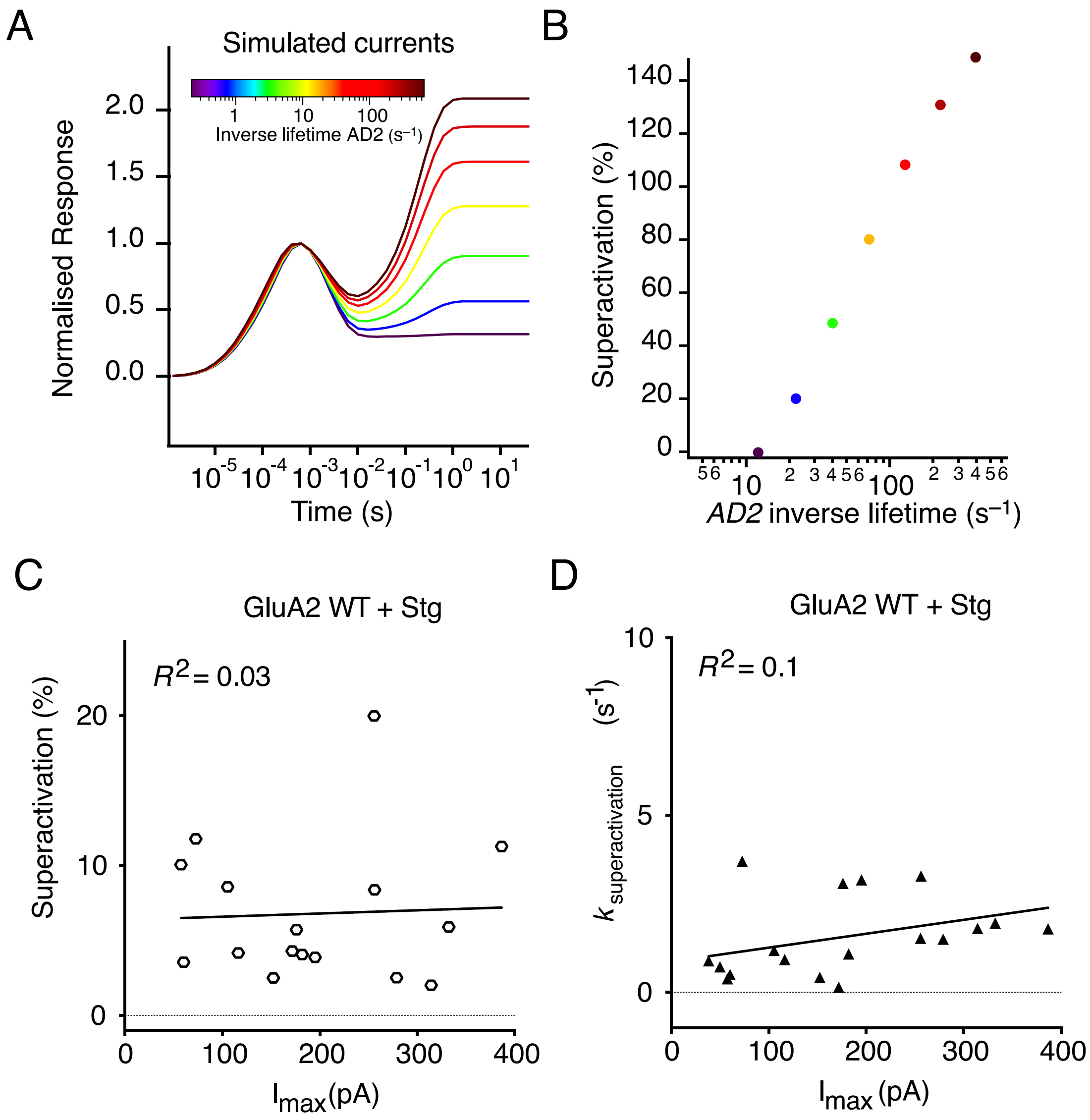

Supplementary Figure 6. Superactivation is correlated to desensitized state lifetime but not apparent channel density. (**A**) When the lifetime of the desensitized state AD2 was varied to produce a range of extents of superactivation, the model from Figure 3B predicted little correlation between rate and extent of superactivation. Note that the abscissa is plotted in log scale. The maximal superactivation is achieved with the same rate in each trace (around 1 s after the initial glutamate pulse). **(B)** The extent of superactivation was strongly correlated to the desensitized state AD2 lifetime. Each symbol represents a simulated trace in panel A. Neither the extent (**C**) nor the rate (**D**) of superactivation was correlated to the peak current measured in outside-out patches, which we take as an index of the density of receptors in the patch.

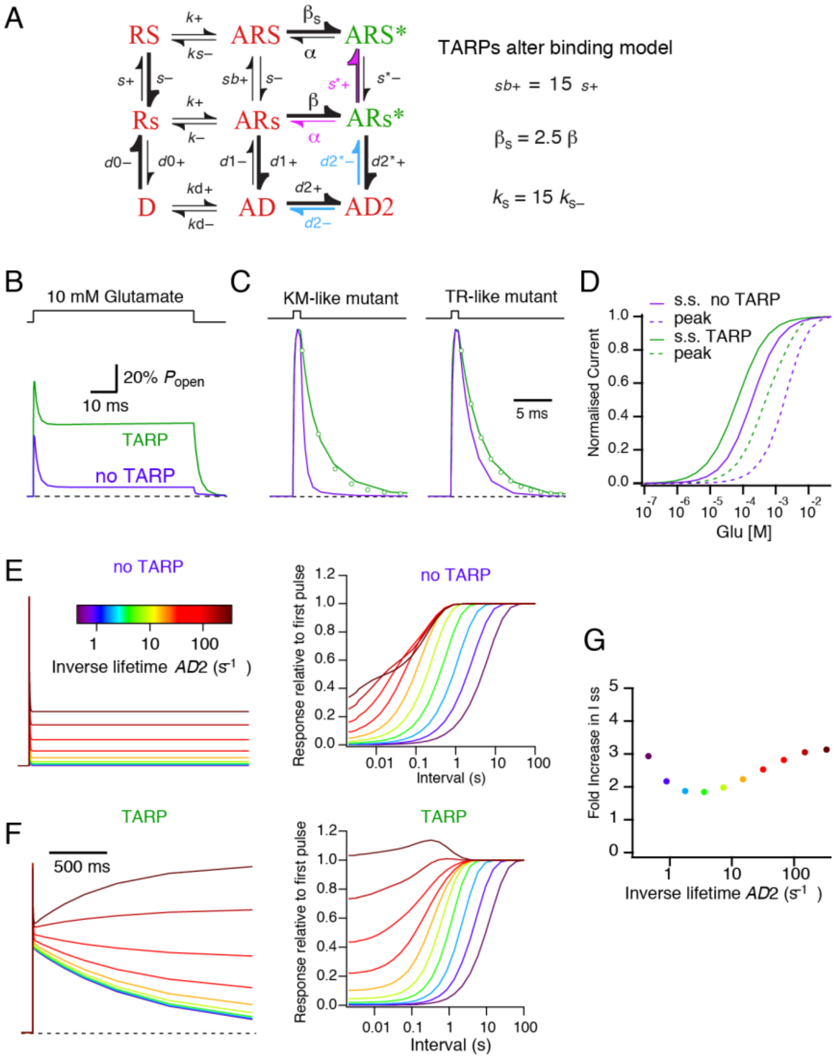

Supplementary Figure 7. A mechanism in which Stargazin chiefly alters glutamate binding. (**A**) Modified version of the model from Figure 3B. Relations between rate constants are indicated. Glutamate was assumed to bind 15-fold tighter in the Stargazin-active state than in the Stargazin basal state. (**B**) The mechanism predicted a larger steady state current but not slower desensitization. (**C**) Deactivation decays were practically monoexponential (fit shown by open circles) for both slow and fast recovering mutants. The experimentally-determined slow component had an amplitude of about 20% for all mutants (data not shown). (**D**) Predicted left shift in the concentration response curve. (**E**) Prediction of long glutamate applications and recovery curves for the model from Figure 3A, mimicking the mutant series without TARPs. (**F**) The TARP binding model predicted muted suprarecovery and slower recovery from desensitization. (**G**) The observed correlation between the fold-increase in steady state current induced by TARP across the mutant series and the recovery time course was not predicted by this mechanism.

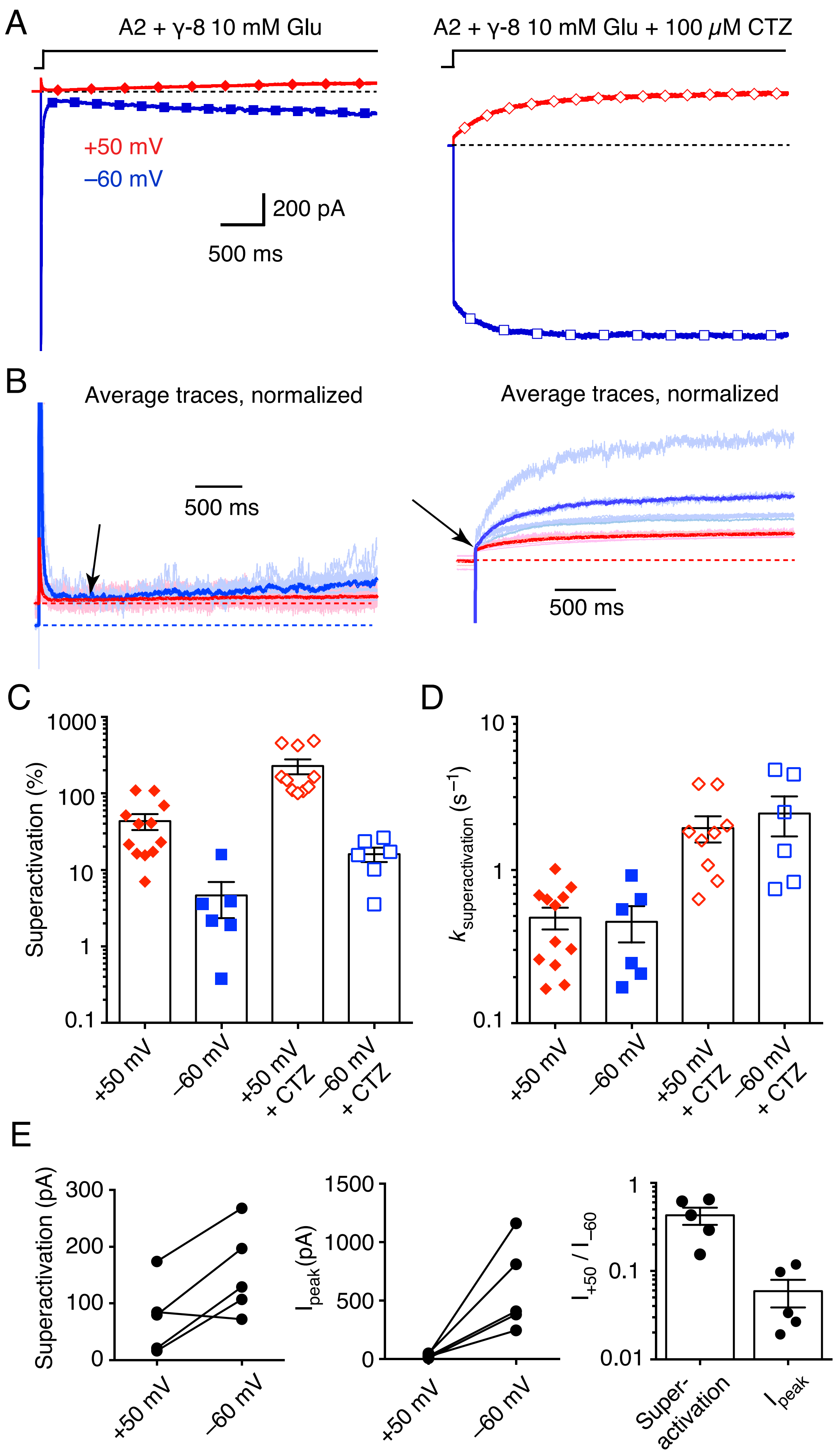
A
A2 + γ-8 10 mM Glu
A2 + γ-8 10 mM Glu + 100 μM CTZ
+50 mV
–60 mV
200 pA
500 ms
B
Average traces, normalized
Average traces, normalized
500 ms
500 ms
C
Superactivation (%)
1000
100
10
1
0.1
+50 mV
–60 mV
+50 mV + CTZ
–60 mV + CTZ
D
k superactivation (s–1)
10
1
0.1
+50 mV
–60 mV
+50 mV + CTZ
–60 mV + CTZ
E
Superactivation (pA)
300
200
100
0
+50 mV
–60 mV
Ipeak (pA)
1500
1000
500
0
+50 mV
–60 mV
I+50 / I–60
1
0.1
0.01
Super-activation
Ipeak

Supplementary Figure 8. Superactivation of GluA2 γ-8 complexes does not depend on voltage. **(A)** Representative traces from the same patch recorded from a cell co-expressing GluA2 and γ-8 at +50 mV (red traces) and –60 mV (blue traces) in the presence (*right*) and absence (*left*) of CTZ. **(B)** Overlay of currents recorded from patches expressing GluA2 + γ -8 in the absence (*left*) and presence (*right*) of CTZ at +50 mV (*blue trace*) and -60 mV (*red trace*). Currents recorded at negative potential have been normalized to the extent of superactivation at +50 mV, recorded in the same patch and condition. Baselines are shifted so as to align the lowest amplitude points after the initial peak (immediately before superactivation becomes apparent, arrows). **(C)** The fraction of superactivation (normalized to the initial peak current) is less at –60 mV, but superactivation could always be measured at negative potentials (43.3 ± 10 % and 4.6 ± 2.3 %, n = 12 and 6, p = 0.012 at +50 mV and -60 mV, respectively). **(D)** The superactivation rate ($k_{superactivation}$) is the same at –60 mV as at +50 mV, either with or without CTZ (0.49 ± 0.08 s^{-1} and 0.46 ±0.1 s^{-1}, n = 12 and 6, p = 0.84 and 1.9 ± 0.4 s^{-1} and 2.3 ± 0.7 s^{-1}, n = 9, p = 0.77, at +50 mV and -60 mV, respectively). **(E)** The absolute magnitude of superactivation in the presence of CTZ is about twice as big at -60 mV than at +50 mV (75 ± 25 pA at +50 mV vs 150 ± 30 pA at –60 mV, n = 5), whereas the peak current was about 20-times larger at –60 mV than at +50 mV (30 ± 5 pA at +50 mV and 600 ± 150 pA at –60 mV, n = 5). Expressed as a ratio, the magnitude of the superactivation currents (comparing +50 mV with –60 mV) was on average 0.43 ± 0.1, whereas the same ratio for initial peak currents was 0.06 ± 0.02. All comparisons were made with a non-parametric randomization test. Error bars represent s.e.m.

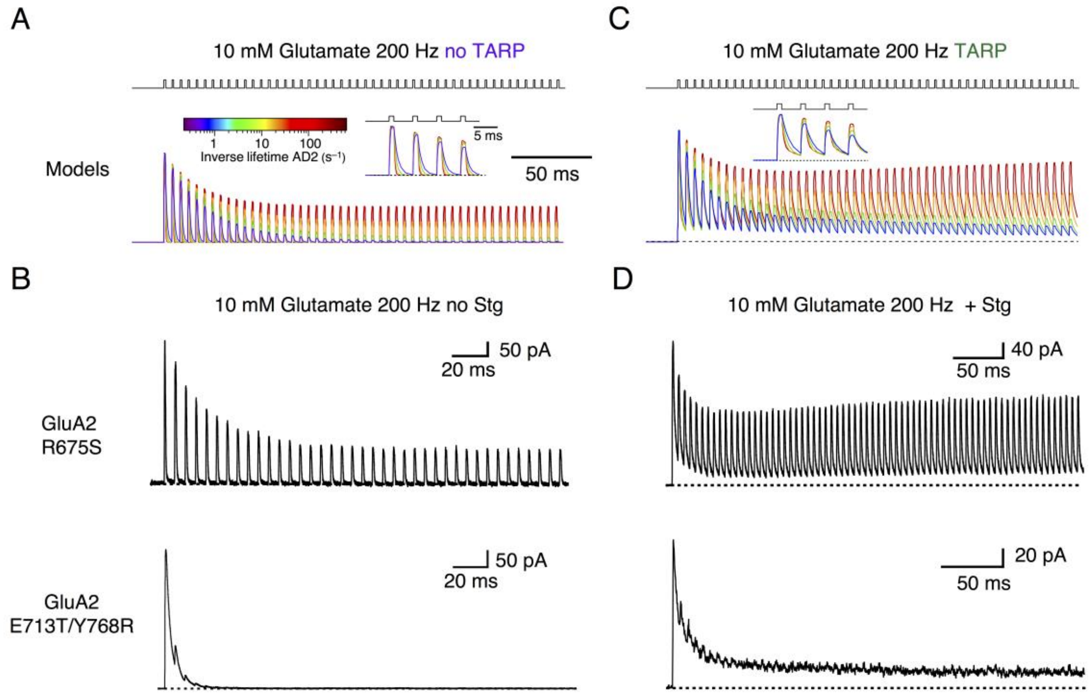

Supplementary Figure 9. Superactivation during trains. **(A)** The model without TARPs (Fig. 3A) predicted that fast and slow recovering mutants depress in response to train stimulation (idealised 200 Hz stimulation, top trace). Color scale indicates the lifetime of the desensitized state AD2 in each simulation. Inset shows the first four responses in the train. Responses were normalized to the first peak. **(B)** All mutants, including the fast recovering mutant GluA2 R675S and the slow recovering mutant GluA2 E713T/Y768R, depressed during 200 Hz trains in the absence of Stargazin. **(C)** The TARP model (Fig. 3B) predicted that slow recovering mutants depress to a steady state current (blue) but fast recovering mutants (including GluA2 WT, red simulated trace) should exhibit superactivation during trains. (**D**) As predicted, in the presence of Stargazin, fast recovering mutants (e.g. GluA2 R675S, upper panel; or GluA2 WT, Figure 7A) exhibited superactivation. Slow recovering mutants (e.g. GluA2 E713T/Y768R, bottom panel) were also consistent with the simulation, depressing slowly to a steady-state current level that was substantially enhanced by the presence of Stargazin.

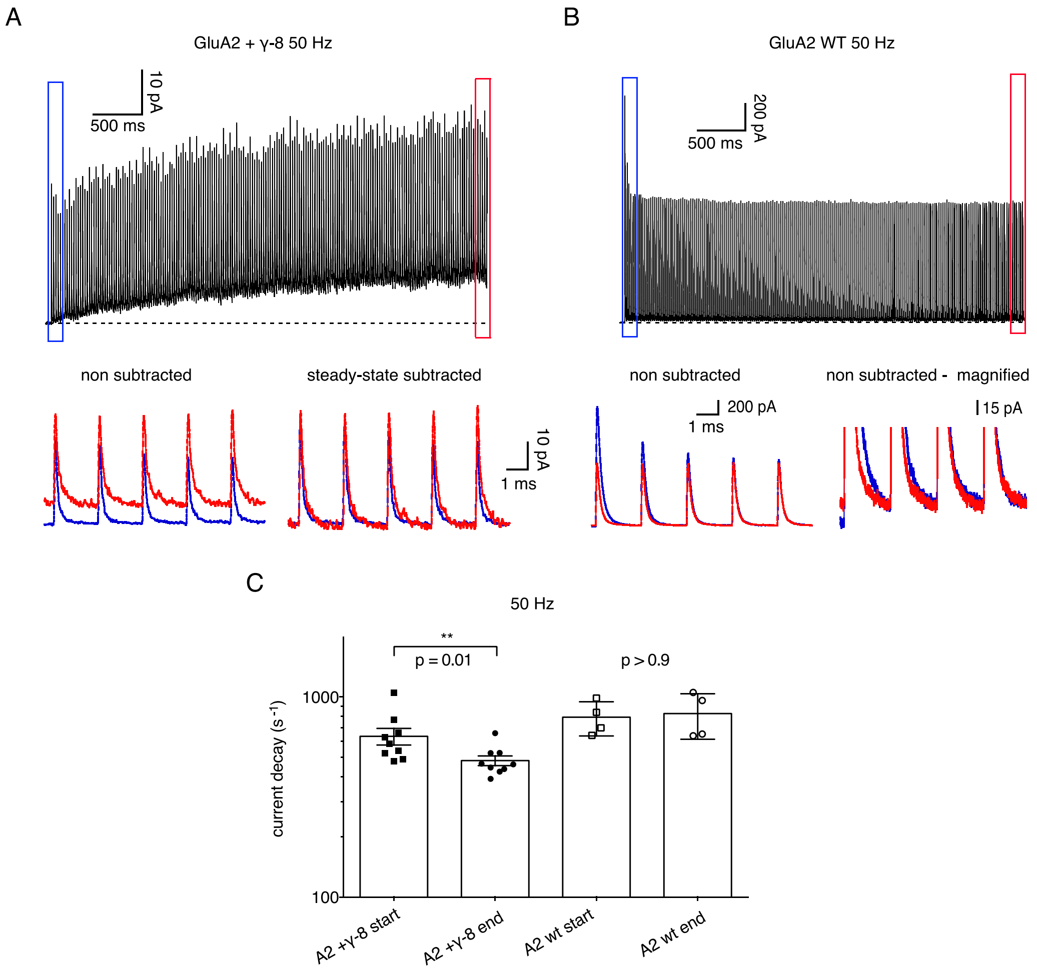

Supplementary Figure 10. γ -8 slows down current decay and increases the standing current during train stimulation. **(A)** γ -8 increases the peak current during 50 Hz train stimulation (*Upper panel*). Overlap of the first five (blue trace) and last five pulses (red trace) shows that the standing current increases during the train (*lower panel, left*). Subtracting the standing current reveals that the current decay is slower at the end of the train (*lower panel, right*). **(B)** Wild type receptors show a decrease in the peak current during 50 Hz train stimulation (*upper panel*). Neither the standing current nor the kinetics of the decay is changed during the train (*lower panels*). **(C)** The decay of the current is significantly slower at the end of the train stimulation for GluA2- γ -8 (n = 9) but remains unchanged for GluA2 alone (n = 4). Error bars represent s.e.m.

.

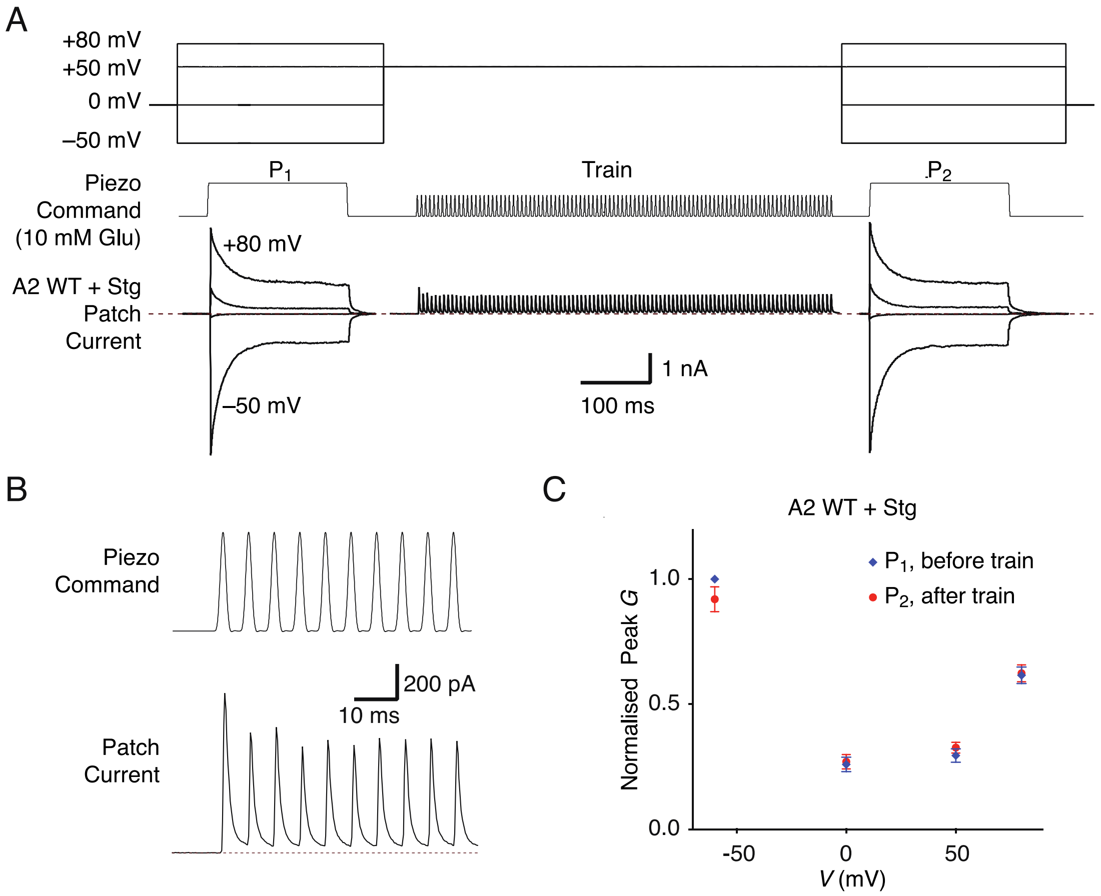

Supplementary Figure 11. Trains that induce superactivation do not reduce polyamine block. (**A**) Voltage protocol (top trace), timing of the 10 mM glutamate pulses (Piezo Command, middle trace) and representative patch currents at the four different voltages. The train of 100 pulses was delivered at 200 Hz. The peak of the post-train response was timed to maximize superactivation. (**B**) Close-up of the piezo command and the 10 responses at the start of the train. (**C)** G-V relations measured before and after train stimulation indicated that superactivation does not relieve polyamine block or change the number of Stargazin molecules associated to the complex during the stimulation (n = 4).

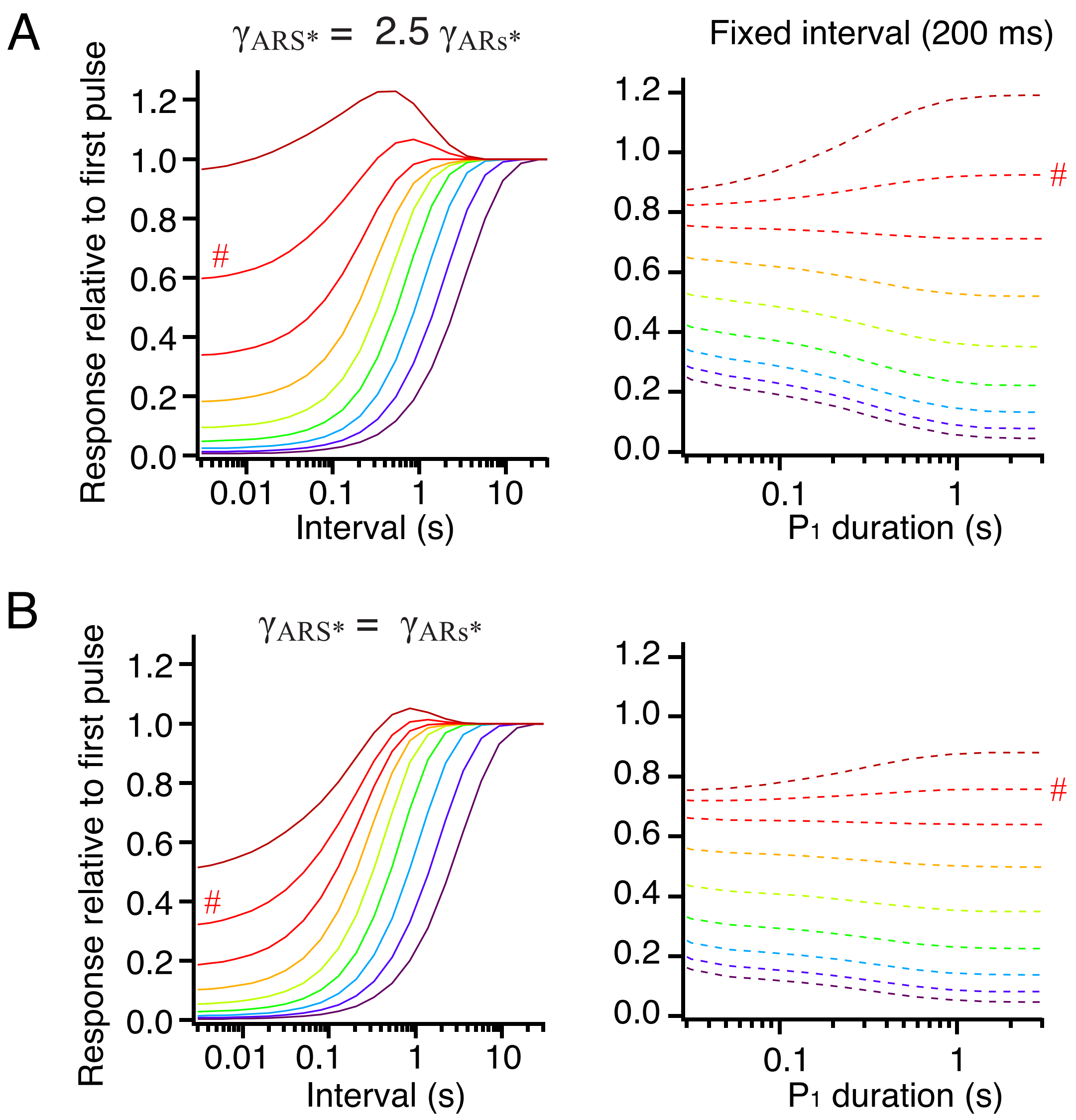

Supplementary Figure 12. Suprarecovery is negligible when the superactive open state has an equal conductance. (**A**) Simulations using the mechanism in Figure 3, with the conductance of ARS* being 2.5 times higher than ARs*. The model predicts that suprarecovery is detectable for WT-like recovery (5%, trace indicated with #) and becomes marked (20%) when recovery is faster (similar to the RS mutant, which had suprarecovery of 22%, Figure 4D). As the length of the first pulse is increased in the range 200-500 ms, suprarecovery increases sharply in magnitude (compare with Supplementary Figure 5C). (**B**) Mechanism from Figure 3, but with the conductance of both open states the same. Suprarecovery is negligible for the set of rates that reproduces wild-type GluA2 kinetics (trace indicated with #). Minor suprarecovery was predicted for the model with recovery faster than wild-type, but only obtained for long pulses of about 1 s or longer, and limited to 5%, about 5-fold less than observed.

Linear Regression in PRISM			
Condition	*Slope*	*95% C.I.*	*P no difference*
A2 + Stg No Spm			
k_{deact}	-0.13 ± 0.33	-1.1 to 0.79	0.71 (*n* = 9)
k_{des}	-0.05 ± 0.02	-0.09 to -0.003	0.04 (*n* = 7)
I_{ss}	0.04 ± 0.02	-0.02 to 0.1	0.11 (*n* = 7)
A2 + Stg + Spm			
k_{deact}	-2.7 ± 0.58	-4.3 to -1.1	**0.009** (*n* = 6)
k_{des}	-0.18 ± 0.03	-0.27 to -0.09	**0.005** (*n* = 7)
I_{ss}	0.10 ± 0.01	0.08 to 0.13	**0.0005** (*n* = 7)
A2-Stg td + Spm			
k_{deact}	-0.75 ± 0.43	-1.9 to 0.44	0.15 (*n* = 6)
k_{des}	-0.02 ± 0.03	-0.09 to 0.05	0.44 (*n* = 6)
I_{ss}	0.06 ± 0.02	0.006 to 0.11	0.04 (*n* = 5)
A2 No Spm			
k_{deact}	-1.4 ± 0.68	-3.3 to 0.52	0.11 (*n* = 6)
k_{des}	-0.03 ± 0.03	-0.12 to 0.052	0.35 (*n* = 11)
I_{ss}	0.005 ± 0.004	-0.006 to 0.02	0.29 (*n* =14)

Supplementary Table 1. Analysis of voltage dependence of kinetics for four conditions shown in Supplementary Figure 1. The very low *p* values (in bold) indicate linear regressions against voltage that are highly significantly different from lines of zero slope. Thus voltage dependence was pronounced in the condition GluA2 + Stargazin in the presence of spermine, as further attested by the higher slope values. In contrast, the linear regressions for the other conditions suggested a lack of voltage dependence, generally indicated by the substantial probabilities of no difference from a line of zero slope and confidence interval spanning zero.

	k_{des} (s^{-1})	k_{deact} (s^{-1})	k_{rec} (s^{-1})	I_{ss} (%)
**A2 WT**	110 ± 5<br>*n* = 31	1250 ± 110<br>*n* = 27	46 ± 3<br>*n* = 14	3.1 ± 0.3<br>*n* = 31
**A2 WT + Stg**	50 ± 2<br>*n* = 43<br>**$p < 0.0000001$**	450 ± 40<br>*n* = 42<br>**$p < 0.0000001$**	53 ± 3<br>*n* = 26<br>$p = 0.18$	30 ± 2<br>*n* = 34<br>**$p < 0.0000001$**
**A2 TR**	130 ± 7<br>*n* = 11	180 ± 25<br>*n* = 6	1.5 ± 0.15<br>*n* = 11	1.1 ± 0.1<br>*n* = 11
**A2 TR + Stg**	115 ± 10<br>*n* = 8<br>$p = 0.22$	131 ± 20<br>*n* = 8<br>$p = 0.11$	1.4 ± 0.15<br>*n* = 7<br>$p = 0.48$	5.4 ± 1.4<br>*n* = 8<br>**$p = 0.0006$**
**A2 ET**	140 ± 12<br>*n* = 7	520 ± 15<br>*n* = 4	16 ± 1.7<br>*n* = 9	1.2 ± 0.3<br>*n* = 7
**A2 ET + Stg**	120 ± 16<br>*n* = 6<br>$p = 0.37$	210 ± 30<br>*n* = 6<br>**$p = 0.005$**	20 ± 1.7<br>*n* = 4<br>$p = 0.10$	5.1 ± 1<br>*n* = 6<br>**$p = 0.0002$**
**A2 YR**	100 ± 7<br>*n* = 8	440 ± 50<br>*n* = 5	14 ± 1<br>*n* = 12	4.5 ± 1.1<br>*n* = 8
**A2 YR + Stg**	55 ± 7<br>*n* = 8<br>**$p = 0.002$**	150 ± 20<br>*n* = 7<br>**$p = 0.0013$**	21 ± 2<br>*n* = 4<br>**$p = 0.0016$**	37 ± 3<br>*n* = 8<br>**$p = 0.0002$**
**A2 RS**	120 ± 8<br>*n* = 8	1500 ± 100<br>*n* = 8	68 ± 9<br>*n* = 8	1.7 ± 0.5<br>*n* = 8
**A2 RS + Stg**	80 ± 7<br>*n* = 12<br>**$p = 0.003$**	380 ± 60<br>*n* = 11<br>**$p = 0.00001$**	71 ± 8<br>*n* = 15<br>$p = 0.86$	40 ± 3.3<br>*n* = 14<br>**$p = 0.000001$**
**A2 KM**	140 ± 8<br>*n* = 9	2300 ± 350<br>*n* = 9	74 ± 7<br>*n* = 11	2.4 ± 0.4<br>*n* = 9
**A2 KM + Stg**	40 ± 2<br>*n* = 16<br>**$p < 0.0000001$**	350 ± 30<br>*n* = 16<br>**$p = 0.000002$**	74 ± 3<br>*n* = 11<br>$p = 0.97$	45 ± 2.2<br>*n* = 16<br>**$p < 0.0000001$**
**GluA2-Stg tandem**	60 ± 3<br>*n* = 23	500 ± 60<br>*n* = 10	43 ± 3<br>*n* = 12	20 ± 2<br>*n* = 23

Supplementary Table 2. Effects of Stargazin on the kinetics of GluA2 WT and mutant receptors. k_{des}, k_{deact}, k_{rec} are the rate of desensitization, deactivation and rate of recovery from desensitization, respectively. See Methods for details of fits. I_{ss} is the steady state current expressed as a percentage of the peak current. Probabilities of no difference (from randomization tests, in bold type where $p < 0.05$) relate to the corresponding wild type or mutant receptor expressed without Stargazin. Currents were recorded at +50 mV in the presence of 50 μM spermine in the pipette solution

for wild-type and mutant receptors cotransfected with Stargazin and at negative potential without intracellular polyamine in the absence of Stargazin.
